# Unraveling the complex enzymatic machinery making a key galactolipid in chloroplast membrane: a multiscale computer simulation

**DOI:** 10.1038/s41598-020-70425-z

**Published:** 2020-08-11

**Authors:** Olga Makshakova, Christelle Breton, Serge Perez

**Affiliations:** 1 Kazan Institute of Biochemistry and Biophysics, FRC Kazan Scientific Center of RAS, Kazan, Russia; 2 University Grenoble Alpes, CNRS, Centre de Recherches sur les Macromolécules Végétales (CERMAV), Grenoble, France

**Keywords:** Biophysics, Computational biology and bioinformatics, Molecular biology, Plant sciences, Structural biology

## Abstract

Chloroplast membranes have a high content of the uncharged galactolipids monogalactosyldiacylglycerol (MGDG) and digalactosyldiacylglycerol (DGDG). These galactolipids are essential for the biogenesis of plastids and functioning of the photosynthetic machinery. A monotopic glycosyltransferase, monogalactosyldiacylglycerol synthase synthesizes the bulk of MGDG. It is embedded in the outer leaflet of the inner envelope membrane of chloroplasts. The protein transfers a galactose residue from UDP-galactose to diacylglycerol (DAG); it needs anionic lipids such as phosphatidylglycerol (PG) to be active. The intricacy of the organization and the process of active complex assembly and synthesis have been investigated at the Coarse-Grained and All-Atom of computer simulation levels to cover large spatial and temporal scales. The following self-assembly process and catalytic events can be drawn; (1) in the membrane, in the absence of protein, there is a spontaneous formation of PG clusters to which DAG molecules associate, (2) a reorganization of the clusters occurs in the vicinity of the protein once inserted in the membrane, (3) an accompanying motion of the catalytic domain of the protein brings DAG in the proper position for the formation of the active complex MGD1/UDP-Gal/DAG/PG for which an atomistic model of interaction is proposed.

## Introduction

Photosynthesis is the process that converts the energy of a photon into chemical energy. Photosynthetic membranes are the most abundant membranes found in nature. Thylakoids are membrane-bound compartments inside the chloroplasts and are the sites of the light-dependent reactions of photosynthesis. They occur in the form of stacks, or grana, made of membranous discs piled one on top of the other, connected by a system of unstacked stroma membranes. The unique lipid composition of photosynthetic membranes remained remarkably preserved during the evolution of cyanobacteria to plants^1^. With a respective distribution of about 50% and 30%, the non-phosphorous and uncharged galactoglycerolipids, namely monogalactosyldiacylglycerol (MGDG) and digalactosyldiacylglycerol (DGDG), compose up to 80% of total lipids of thylakoid membranes^2^. The remaining lipids consist of the anionic sulfoquinovosyldiacylglycerol (SQDG) and phosphatidylglycerol (PG)^3^. In addition to having unusual head groups, MGDG and DGDG contain two highly unsaturated fatty acid chains, which give them unique packing properties. The major galactolipid MGDG, due to its conical shape, tends to adopt the hexagonal type II (inverted hexagonal, H_II_) structure. The three other lipids DGDG, PG and SQDG have a cylindrical shape and pack as a bilayer^4–6^. The structural specificities of galactolipids contribute to the organisation of thylakoids and the functional distribution of photosystems along thylakoids. Photosystem 1 (PSI) is highly enriched in stromal membranes, and photosystem II (PSII) is concentrated in stacked membranes^7^.

The property of MGDG to form H_II_ phases could be beneficial in highly curved membrane domains or near large protein complexes. Indeed, the coexistence of bilayer and non-bilayer phases in photosynthetic membranes may regulate the protein content and activities of certain enzymes. It was shown, for instance, that the violaxanthin de-epoxidase requires an H_II_ phase for proper functioning^8,9^. On the opposite, some membrane proteins, such as the Light-Harvesting Complex (LHC-II) of PSII, may force MGDG to adopt a bilayer structure^10^. Maintaining a constant MGDG/DGDG ratio in thylakoid membranes is, therefore, considered crucial for the structure and stability of photosynthetic membranes^11^. This ratio is regulated in response to different stresses to maintain membrane structures and enzymatic activities^12^. Studies have shown that membranes reconstituted from lipid extracts of natural thylakoids can self-organize into membrane bilayer. They can pass, in a reversible way, from the H_II_ phase to the lamellar phase by playing on the respective concentrations of the lipid species and in particular, on the MGDG/DGDG ratio. This phase transition also depends on the level of hydration, which can be influenced by the protein composition of the membrane^13^. In regards to the several roles of MGDG in the biogenesis and architecture of chloroplast membranes of higher plants, the understanding of molecular aspects of its synthesis is needed to provide fundamental knowledge on this process and to open perspectives for the construction of artificial chloroplast. The current research is addressed to the elucidation of the mechanisms of the assembling of the active synthetic complex making MGDG on the membrane surface.

**Figure 1:**
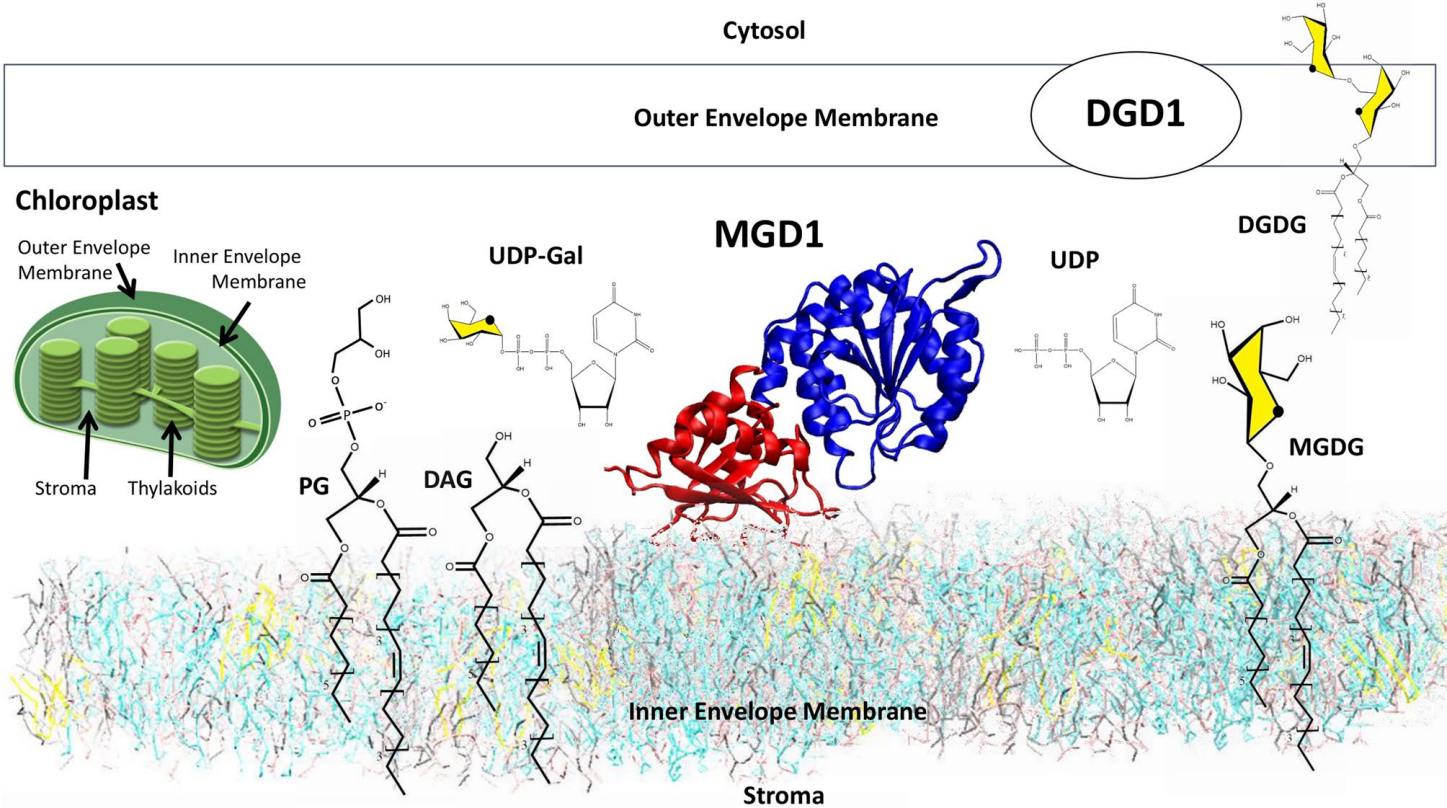
Schematic representation of the main actors for the synthesis of monogalactosyldiacylglycerol (MGDG) in the Inner Envelope Membrane (IEM) of the chloroplast. The N- and C-domains of MGD1 are highlighted in red and blue; respectively, the galactosyl residue is shown in yellow.

In Arabidopsis, the monogalactosyldiacylglycerol synthase 1 (MGD1) is the primary enzyme responsible for the synthesis of the bulk of MGDG. MGD1 is a monotopic membrane protein localised in the inner envelope membrane (IEM) of chloroplasts^14^. It transfers one galactose residue from the water-soluble donor substrate, UDP-α-d-galactose, to the hydrophobic acceptor substrate, diacylglycerol (DAG), to form MGDG (Galβ-DAG) (Fig. 1). Once assembled, MGDG is transferred by a yet unknown mechanism to the outer envelope membrane, where DGDG synthesis occurs, and to the nascent thylakoids. The flux of DAG, the direct precursor of MGDG, must be tightly controlled to meet the galactolipid demand during the rapid and massive expansion of photosynthetic membranes upon light illumination. The content of DAG is very low in the chloroplast membrane (less than 1 mol%), suggesting that it is rapidly converted into MGDG once formed. The question remains as to elucidate the mechanisms of such an efficient recognition of DAG by MGD1.

The peculiar orientation of MGD1 toward the membrane surface should favour the capture of both the lipidic DAG substrate and the soluble UDP-Gal substrate. Recently, an X-ray diffraction study established the spatial structure of MGD1 in non-bound form and bound with the UDP moiety^15^. MGD1 adopts the canonical GTB fold consisting of two separate Rossmann-type domains of similar size connected by a large cleft forming the active site^15^. Based on structural data, a model of protein–membrane interaction was proposed in which the protein is attached to the lipid membrane via a large and flexible region (herein called the LOOP) in the N-terminal domain. The LOOP region (~ 50 amino acids) is essential for the capture of DAG, whereas the C-terminal domain is mostly involved in donor sugar-binding^15^. The histidine residue, H155, acts as the catalytic base that deprotonates the hydroxyl group of DAG acceptor. The structure of the large LOOP remained unassigned due to its high flexibility and disordering. Besides hydrophobic and hydrophilic substrates, MGD1 needs anionic lipids such as phosphatidic acid (PA) or PG to be active^16^. Recent data gave insight into the mechanism of MGD1 activation by PG. The activator binds in a region close to the DAG binding site suggesting the existence of a PG-His catalytic dyad^17^. In contrast, PA is expected to proceed through a different mechanism since it exhibits an allosteric behaviour (not observed in the case of PG)^16^.

One way to study the peculiar role of different lipids in MGD1 functioning, and to decipher the way MGD1 binds to the membrane is to use in vitro monolayers of lipids of simplified and variable composition. Recent studies have indicated visible preferences of MGD1 to certain lipid species^15,17,18^. Notably, MGD1 demonstrated a high affinity to MGDG (its reaction product), PG, and DAG monolayers, but not to DGDG. Interestingly, the addition of PG overcomes the negative effect of DGDG on MGD1 binding^18^. These facts all together allow assuming the existence of spontaneous or/and induced nano-domain organisation of the complex IEM. Such domains could serve as a platform for MGD1 to bind and to perform its catalytic action.

The present work aims at studying the interactions between MGD1 and the most representative biomimetic lipid bilayers at the atom level, with a particular focus on lipids needed for MGD1 activity (PG and DAG) and their clustering in the presence of MGDG and DGDG. Studying the structure and dynamics of lipids, protein, and carbohydrates is challenging. Relevant length and time scales are not easily accessed experimentally. Because of the size of the macromolecular systems in interaction, we applied the Coarse-Grained (CG) molecular dynamics (MD) approach to unravel the main features of interactions between MGD1 and the lipid bilayer and the lipid capture. Further, All-Atom (AA) simulations of the MGD1/lipid bilayer system were applied to elucidate a possible influence of membrane surface on intramolecular protein dynamics. The explicit consideration of hydrogen bonds contributed to shedding light on the possible ways of the regulation of protein activity. The CG MD simulations are powerful and useful tools to explore the architecture and the dynamical organisation of complex lipid membranes. CG simulations have been widely applied to both single membranes, including plant thylakoid and mammalian plasma membranes^19,20^ and membrane-protein systems^21,22^. They helped to unravel the role of complexity and crowding in the functioning of proteins embedded into or working on the membrane surface. The CG Martini representation of a (macro)molecule that groups four non-hydrogen atoms in a single bead provides a unique way to simulate larger molecular systems. While reducing the cost of computation without sacrificing molecular details, CG simulation yielded some relevant information that relates to experimental. The capture of the long temporal scale and molecular representation CG can fill the gap between experiment and AA simulations.

## Results

### Membrane composition and characterization

We investigated four compositions of the membrane listed in Table 1. Model M1 was composed only of PG and DAG because these two lipids are necessary and sufficient to probe MGD1 activity while interacting with the membrane^17^. In Model M2 galactolipids MGDG and DGDG were added to PG and DAG in the respective composition that reflects the IEM lipid composition in terms of MGDG, DGDG and PG^2^. The MGDG and DGDG have, for the most part, polyunsaturated fatty acid tails^13^ while the tails of PG and DAG were mostly unsaturated. Models M3 and M4 are close to M2 with slight changes in the proportions of lipids. Model M3 has an accumulative amount of anionic lipids the closest to that in IEM^2^. In Model M4, a slight change brings the composition closer to the one used for the simulations of thylakoid membrane^19^, but which did not consider the presence of DAG.

**Table 1:** Characteristics of the lipid composition in the model membranes.

	M1 (%)	M2 (%)	M3 (%)	M4 (%)
MGDG		50	50	40
DGDG		30	30	30
PG	75	10	15	25
DAG	25	10	5	5

Given the uniqueness and complexity of the lipid composition of the membrane, the critical point is to establish the adequacy of the computational procedure and compare the results with either previous similar investigations or available experimental data. All the calculations performed on membranes having xy-dimensions of 8 × 8 nm showed no significant membrane undulations.

Among the four compositions studied, the one corresponding to model M1 yielded a bilayer having the largest thickness of 3.4 nm (see Table 2). This is per its large proportion of saturated lipid tails. Molecular flexibility of a lipid is associated with the saturation of its hydrocarbon chains. Unsaturated and, in particular, polyunsaturated lipids are more flexible and are expected to partition to curved regions to lower the energetic cost of membrane deformation. More rigid saturated lipids which display a tight packing are depleted from curved areas^23^. The values of surface area (SA) per lipid, which is the lowest in model M1, increases in other models as M4, M3 → M2, and the module of compressibility (KA) decreases in the range M1 → M4 → M3 → M2. Such a trend reflects the influence of the number of unsaturated lipid tails. Model M4, whose composition relates closely to that used for simulations in Ref.^19^, agrees with the ones reported previously for plant thylakoid membrane at both CG and AA levels of representation^19^.

**Table 2:** Structural and dynamical properties of model membranes: surface area per lipid (SA), compressibility modulus (KA) and membrane thickness (d), lateral diffusion coefficient (D) and flip-flop frequency calculated at CG resolution.

	M1	M2	M3	M4	Thylakoid, CG^19^	Thylakoid, AA^19^
**Small membrane**						
SA, nm^2^	0.58 ± 0.01	0.65 ± 0.02	0.63 ± 0.01	0.63 ± 0.01	0.66 ± 0.001	0.66 ± 0.003
KA, mN/m	619 ± 7	141 ± 3	360 ± 5	363 ± 5	240 ± 16	311 ± 125
d, nm	3.4 ± 0.1	2.9 ± 0.1	2.9 ± 0.1	2.9 ± 0.1	2.9 ± 0.001	2.8 ± 0.02
**Large membrane**						
D, µm^2^/s						
MGDG		32.3 ± 0.6	20.9 ± 1.5	23.2 ± 0.1	32–34	
DGDG		31.9 ± 0.3	21.5 ± 0.5	24.6 ± 1.2	28–32	
PG	35.6 ± 3.7	0.6 ± 0.1	14.9 ± 2.8	15 ± 1	19–34	
DAG	40.8 ± 0.4	3.5 ± 0.2	6 ± 4.7	5.8 ± 3.6		
Flip-flop of DAG, µs^−1^	1 ± 0.4	10 ± 2	6 ± 2	4 ± 1		

The mean values and standard errors were estimated along the last 1 µs of corresponding trajectories.

**Figure 2:**
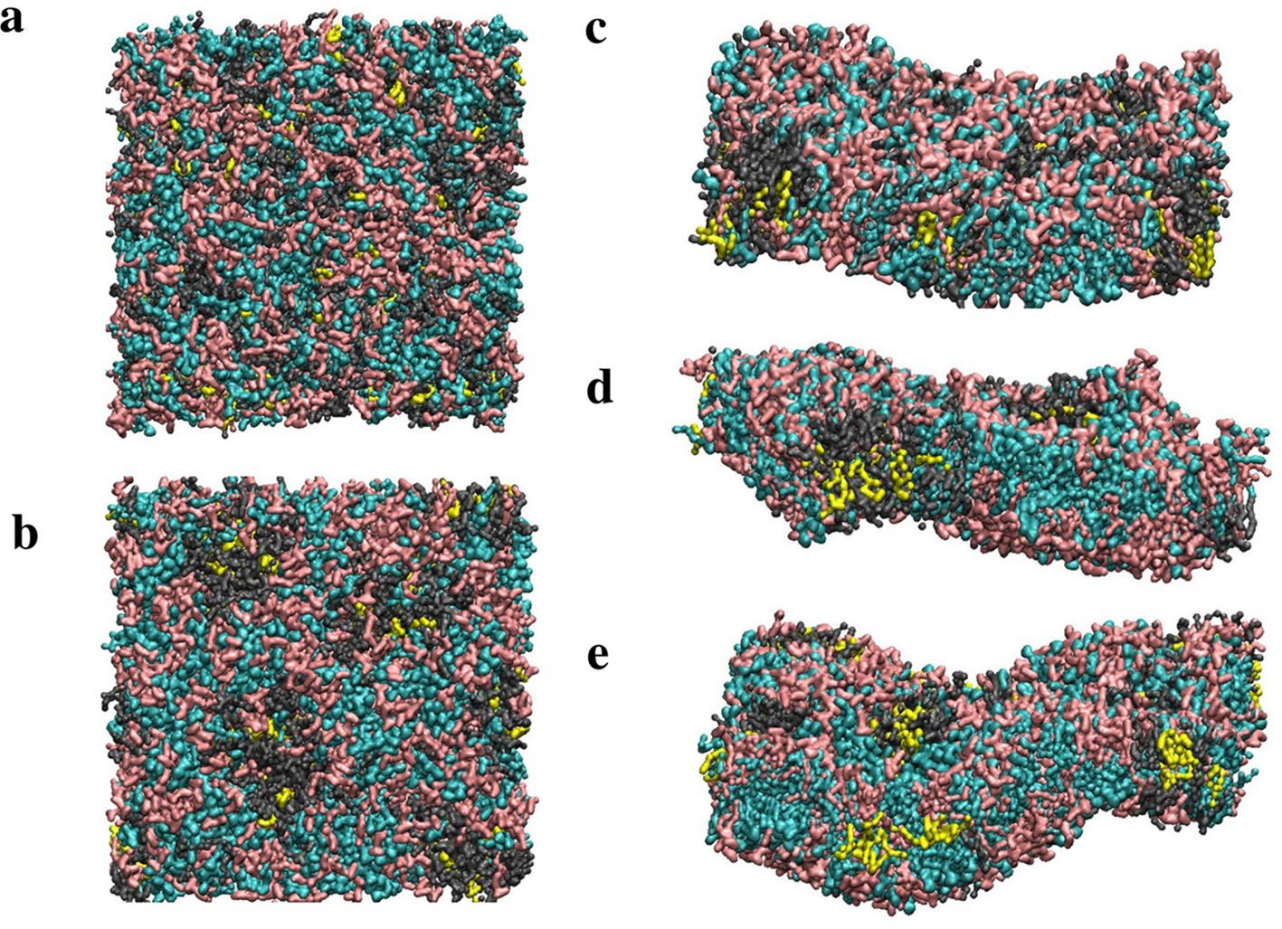
The top view of M4 membrane before (**a**) and after (**b**) equilibration in the course of CG MD trajectory and the side view showing the curvatures of M4 (**c**), M3 (**d**) and M2 (**e**) membrane models (size 20 × 20 nm). Colour coding is as follows: cyan is for MGDG, pink for DGDG, grey for PG and yellow for DAG.

The extension of membrane size up to 20 × 20 nm (the size of the unit cell of the periodical box) led to marked undulations of the membrane surface in some models (Fig. 2). The M1 bilayer remained almost flat (not shown). The increasing number of lipids with unsaturated tails led to an increase of the membrane curvature in the range M4, M3 → M2 (see density profiles for GL1 beads of each species in Supplementary Fig. S1.1).

The long-term diffusion constants calculated from the mean squared displacement of the lipids of different species (Table 2) provide a way to evaluate the lateral diffusivity of the lipids. The diffusion constants of MGDG and DGDG, in the three membrane models (M2–M4), lay in approximately the same region. The constants for M2 fit well with those predicted for the plant thylakoid membranes^19^. Taking into account the fact that CG representation of lipids speeds up the diffusion due to smooth energy landscape, the values of the diffusion coefficient should be divided by a factor about four to five^24^. An intriguing observation is that the lateral diffusion coefficients for both PG and DAG become significantly lower in bilayers M2, M3, and M4 than in PG/DAG (M1) model. As constituents of bilayers M2, M3, and M4, PG and DAG molecules have a much lower lateral diffusion coefficient than those of the glycolipids MGDG and DGDG. This fact seems to result from the differences of mixing between those lipids with mostly polyunsaturated tails, and those with mostly saturated tails.

**Figure 3:**
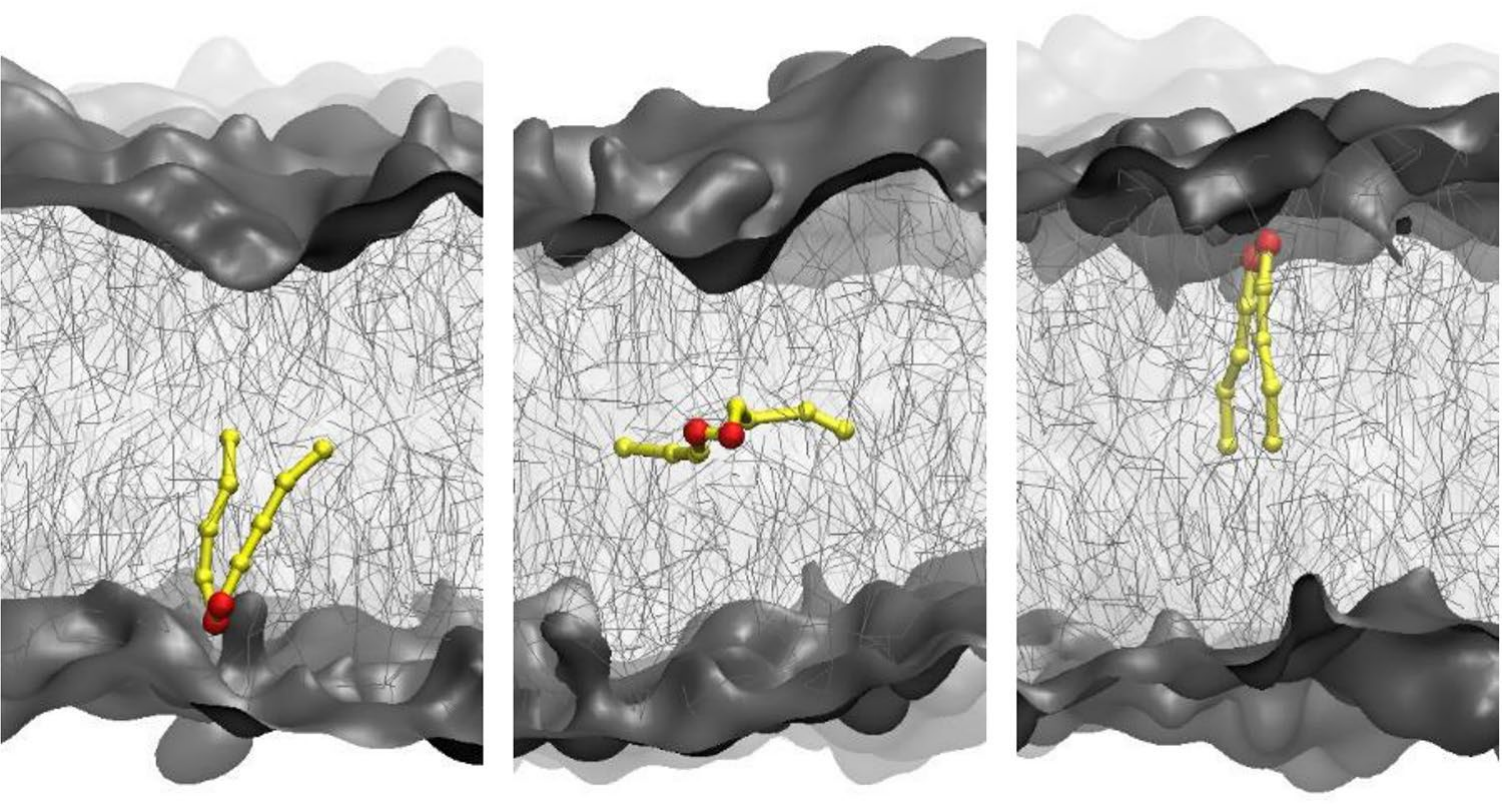
The transverse diffusion of DAG molecule from one to the other leaflet crossing the hydrophobic core of the lipid membrane.

Furthermore, the loose packing of the glycolipid bilayers provides favourable conditions for DAG molecules to flip within the membrane (Table 2). The number of events of transverse diffusion of DAG molecules increases in the range M1 → M4 → M3 → M2. On average, one flip-flop of DAG molecule was detected in M1 per 1 µs while about four to ten spontaneous flip-flop transitions occur in the presence of galactolipids (Fig. 3). For comparison, in the plasma membrane, both cholesterol and DAG molecules demonstrated around six flip-flops per µs^20^. The DAG molecule is small compared to glycolipids (MGDG and DGDG) and PG. Not having any bulk hydrophilic head groups, DAG molecules tend to hide from water in the hydrophobic core^25^. This facilitates natural flip-flop motions. In comparison, no events of flip-flopping for galactolipids or PG were detected during the trajectories.

In the model membranes, both visual inspection (Fig. 2) and evaluation of the percentage of contacts between lipid molecules of different species demonstrate the tendency to form clusters (Supplementary Table S1.1). In the model M1, the lipid species are well mixed (67% and 20% self-contacts of PG and DAG molecules as compared to the 75% and 25% expected for a uniform distribution). Whereas, in the glycolipid membranes, MGDG shows a slightly higher percentage of self-contacts (by ~ 10% from those expected from the random distribution, namely 50% for M2 and M3 and 40% for M4). The rate of self-contacts for DGDG species is slightly decreased in all galactolipid models compared to those expected from a random distribution. The striking observation is that both PG and DAG moieties demonstrate a pronounced increase of self-contacts and contacts between the counter-part species (two to three times more than expected from equal distribution). The increase of the number of such interaction occurs at the expense of the number of contacts with both MGDG and DGDG. This highlights the formation of PG/DAG clusters with an average number of 5–11 lipids per cluster. The occurrence of PG nano-sized clusters agrees with the results of simulations reported in Ref.^19^. The existence of such clusters explains the reduced lateral diffusion and increased transversal diffusion as compared to more rigid PG/DAG bilayers.

For the further analysis of the influence of MDG1 on membrane properties, the M4 bilayer was used for chloroplast mimicking, because it revealed less pronounced flexibility. The size of the membrane with xy-dimensions of 20 × 20 nm was considered significant to study its interactions with MGD1, whose longest dimension is around 8 nm.

### Full-length model of MGD1 construction

The elucidation of the crystal structure of MGD1 failed to reveal a vast and disordered region of nearly 50 amino acid residues in the N-domain that was attributed to crystallographic disorder^15^.

The application of the i-TASSER procedure for the de novo construction of the LOOP yielded five likely models of the entire MGD1 protein. Among the initial models, some presented similarities as assessed by the magnitude of the root mean square deviation. Those being below a value of 0.6 nm were discarded. Supplementary Fig. S2.1 shows the most structurally distinct models (Model1, Model4 and Model5 numbered according to i-TASSER output files). In a second step, the calculation of the Normal Mode Analysis [in the framework of Anisotropic Network Model (ANM)^26^] provided the per-residue B-factors. The ANM predicts anisotropic motions on the base of the orientation of interactions between nodes, centered on Cα atoms, for the global coordinates considering the overall potential as the sum of harmonic potentials between interacting nodes. The low-frequency collective motions were analyzed. Two models (Model4 and 5) displayed large motions for the LOOP (Supplementary Figs. S3.1–S3.3). However, in Model1, the predicted motions of the LOOP are limited (Supplementary Fig. S3.1), with the calculated B-factors being close and even lower than those experimentally reported. In this model, the interactions between the LOOP and the rest of the protein occur in the vicinity of the inter-domain cleft, which hardly can remain catalytically active. Such a variety of MGD1 models points towards the pronounced flexibility of the LOOP and may explain the lack of observed electron density in the crystal structures^15^.

#### Protein dynamics in water

The refinement of the 3-dimensional structure of the predicted models requires further computational steps, using AA MD simulations. Supplementary Fig. S3.5 summarizes the main results derived from an AA MD performed for a 600 ns trajectory for the two best models (Model4 and Model5). In short, the analysis indicates that the root-mean-square fluctuations (rmsf) of Cα residues in the water box are well matched for both models (see Supplementary Fig. S3.5). Moreover, the calculated rmsf values are in good agreement with the experimental B-factors (Supplementary Fig. S3.6). Starting from two protein structures with the extreme positions of LOOP, the structures merge in the course of trajectories covering the conformational space of the protein. Probing the high flexibility of the LOOP in silico, we cannot prefer Model4 over Model5 (or intermediate conformations) at the current step of full-length protein modelling. The interactions with the lipid membrane will determine the eventual LOOP conformation of active enzyme. To this end, both implicit and explicit membrane was consequently taken into consideration. The knowledge-based constraints were used to select a model. Namely, those LOOP residues that, according to mutagenesis experiments, were crucial for MGD1 activity were assumed interacting with lipid moiety^15,17^.

#### Protein–membrane pre-orientation

The procedure of pre-orientation of MGD1 to the membrane surface was based on the minimization of the energy of protein transfer from water to the membrane^27^. The results supported the monotopic localization of the MGD1 enzyme (shown in Supplementary Fig. S4.1). For the three models considered, Model1, Model4 and Model5, the depth of burying varied significantly from 1.6 to 6.1 Å. Such interactions led to the transfer of energy gain from − 3.2 to − 5.6 kcal/mol (Supplementary Table S4.1). The most significant energy gain occurred when the protein interacted with the membrane surface by the LOOP, as in case of Model4 and Model5. Model4 shows the largest energy of transfer to the membrane and the most probable LOOP orientation, which determines the spatial location of P189 buried into the membrane. This residue is expected to interact with PG^15,17^. The LOOP orientation in Model4 goes from the equilibrated complex with the explicit membrane, where P189 and catalytic H155 participate in the formation of a surface of the binding site of PG and DAG (more details will be given in the next section). On the contrary, the P189 was exposed to the solvent after the equilibration of Model5 placed at the explicit membrane. Hence, Model5 was discarded as less likely conformer for the formation of the active complex between MGD1, and the membrane and Model4 were selected for further analysis.

### Protein–membrane interaction and capture of PG and DAG

The protein–membrane simulations were carried out at CG-representation, which allows covering large timescales relevant for such a complex system equilibration. The CG Martini force-field has already been used for CG simulations of lipid membranes. Examples include thylakoid membranes of higher plants^19^ and transmembrane protein–thylakoid membrane interactions^21^. These previous simulations provided consistent results with those obtained at the AA level, and those obtained experimentally.

The CG model of MGD1 was used without any restraints (see Supplemental Information 3). For the trajectories, the flat bilayer model M4 (MGDG/DGDG/PG/DAG, 40/30/25/5) with a random lipid distribution was taken as the initial state. The MGD1 (Model4) was pre-oriented with respect to the membrane, as reported above.

In the course of system equilibration, the collective vibrations of bilobal protein, particularly slow screwing and twisting motions in the linker connecting two domains, resulted in the alteration of protein orientation with respect to the membrane plane. SI movies display an animation of protein movements along PC1 and PC2 in the presence of membrane; they are slightly different from those corresponding for protein without a membrane (Supplementary information S3.3). When the membrane becomes incurved, the protein is located on the slope of membrane wave with N-domain placed on the bottom of the curve. The curved membrane surface helps the protein to establish the interactions with the residues of N-domain and a part of C-domain while bringing the MGD1 active site close to the membrane surface.

**Figure 4:**
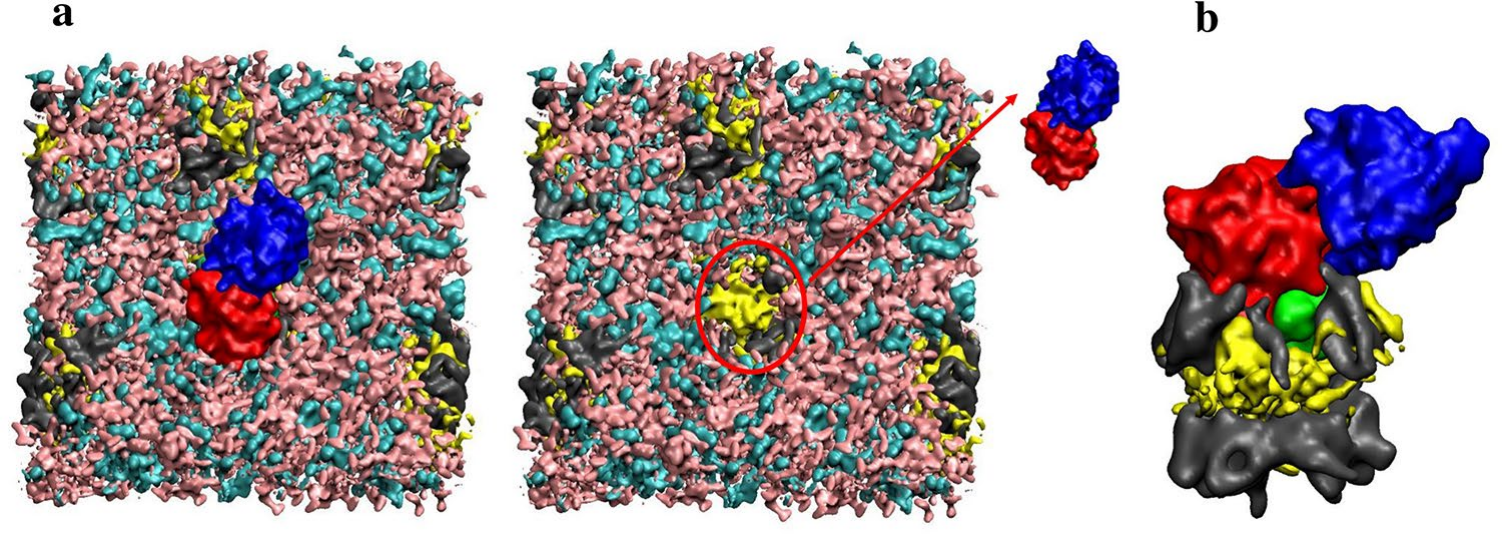
A snapshot of MGDG(cyan)/DGDG(pink)/PG(grey)/DAG(yellow) M4-bilayer and MGD1 (N-domain is in red, C-domain is in blue, LOOP is in green), the view from the top (**a**). The PG/DAG cluster with MGD1 bound, the frontal view (**b**).

In the presence of protein, the tendency of PG and DAG to form nano-sized clusters was observed similarly to that described in the absence of protein. The timescale of such a cluster formation was around a dozen nanoseconds. After an equilibration period of 100 ns, the protein revealed a tendency to locate in the vicinity of such PG/DAG clusters. The interactions of MGD1 with PG and DAG occurred mainly via the residues of N-domain, particularly those in the LOOP region. Figure 4 illustrates the orientation of MGD1 concerning the membrane after 500 ns equilibration. Figure 4b demonstrates that the size of the protein suits perfectly with the size of the PG/DAG cluster. A fascinating observation is an additional accumulation of DAG molecules in the vicinity of MGD1 when compared to the clusters formed without protein (Fig. 4a, see also Fig. 2b). In such clusters, the number of contacts of MGD1 with DAG molecules is larger than the number of contacts with PG (Supplementary Table S5.1). Such an accumulation can occur due to the favourable interactions of specific lipids with MGD1 and driven by both lateral and transverse diffusion of DAG molecules, which is facilitated in galactolipid membranes.

**Figure 5:**
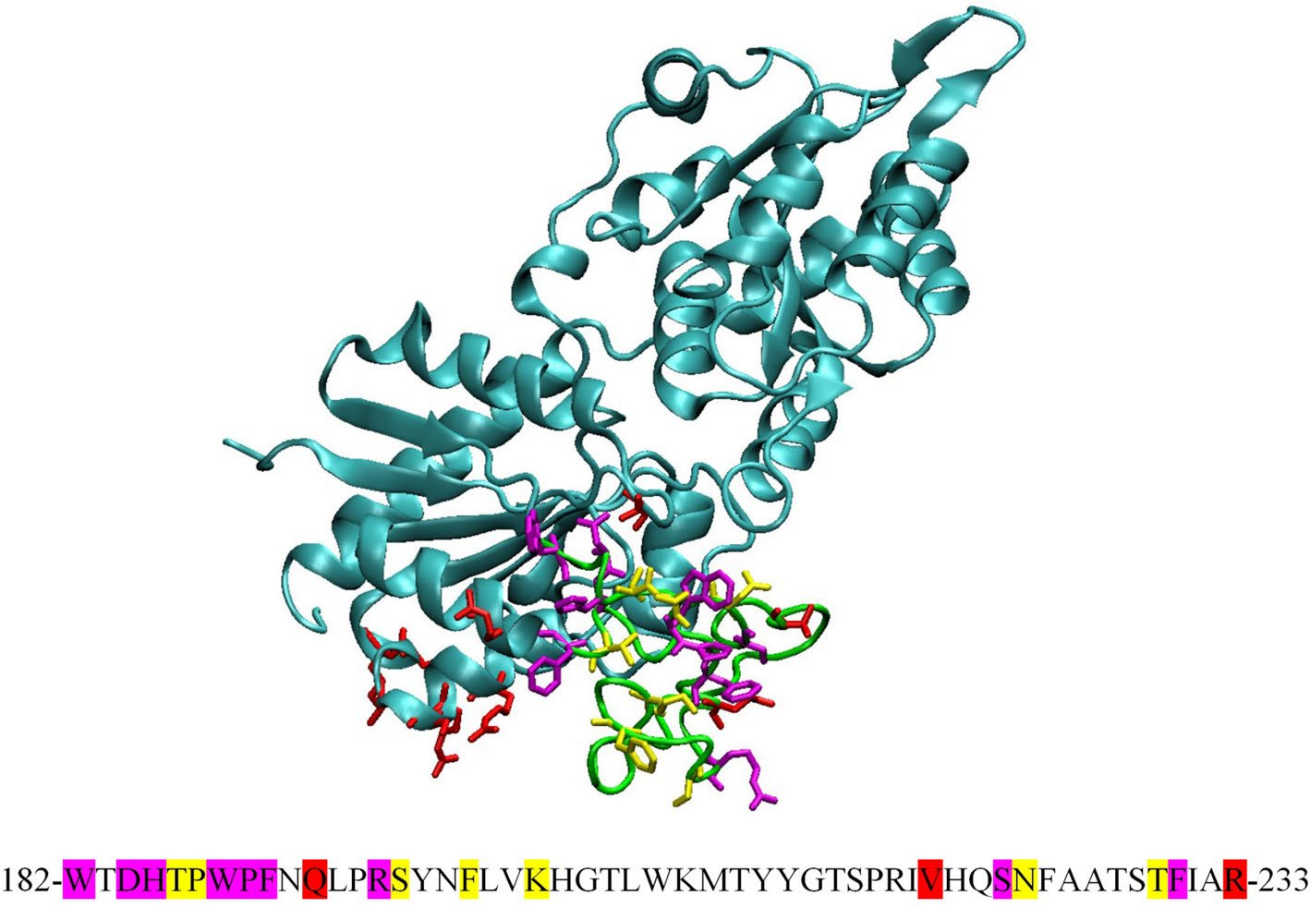
The structure of MGD1, LOOP indicated by green colour. Residues that interact preferably with DAG or PG molecules on the averaged distance less than 0.8 nm (a size of first hydration shell for Martini) are shown as sticks and highlighted in yellow and red, correspondingly. Those interacting with both DAG and PG are in magenta. The same colour coding is used to indicate corresponding residues on the LOOP sequence.

**Figure 6:**
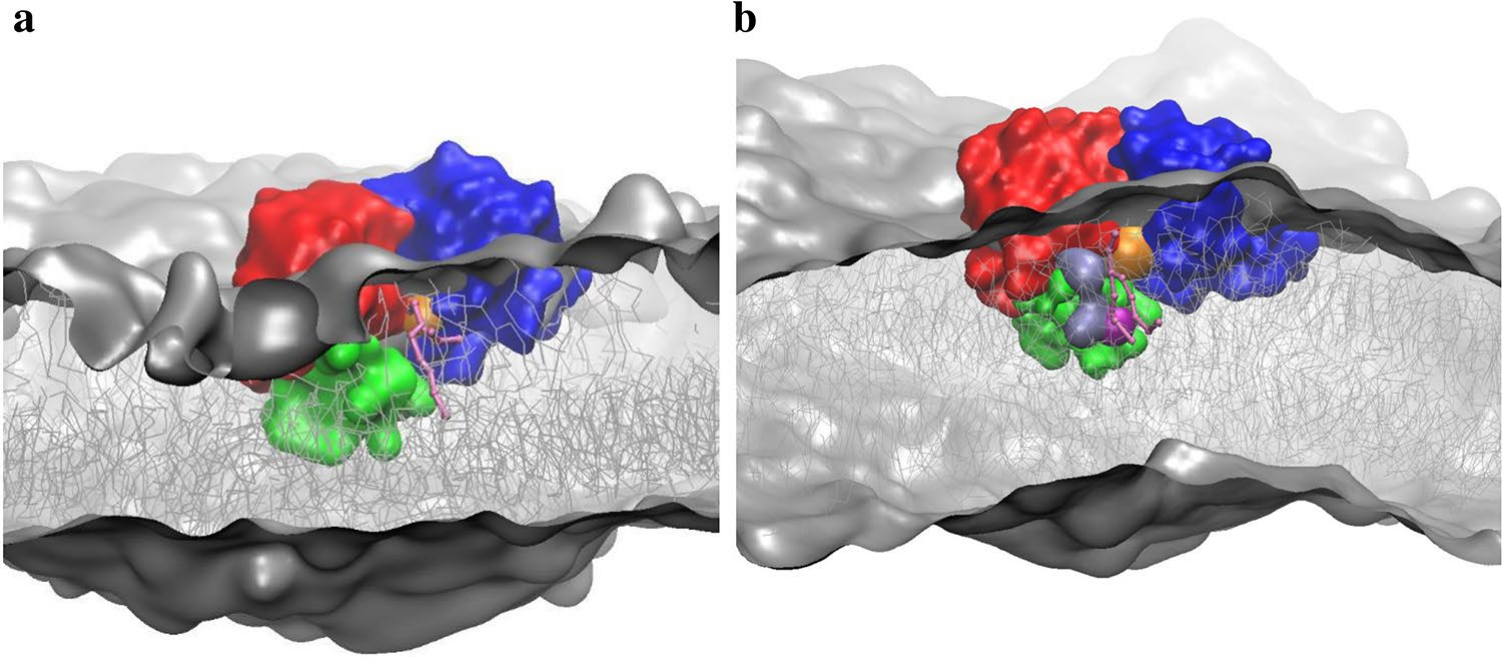
Snapshots of MGDG/DGDG/PG/DAG membrane and MGD1 protein (N-domain is in red, C-domain is in blue, LOOP is in green, catalytic H155 is in orange, PG is in pink). The PG molecule interacting with the catalytic residue is shown as balls and sticks (**a**). The PG molecule is interacting with residues of the LOOP (indicated in ice-blue, P189 in magenta).

The location of MGD1 in the vicinity of PG/DAG clusters should facilitate the capture of the PG and DAG molecules. Supplementary Tables S5.2 and S5.3 contain the list of critical residues involved in the interaction of MGD1 with DAG and PG, respectively. The interactions with DAG mostly occurred via residues of the LOOP region in the vicinity of the active site (see Fig. 5). There are many aromatic and non-polar residues among the residues having the tightest contacts with DAG (the averaged over MD trajectory distance between the residue and any part of lipid moiety less than 0.8 nm, taken as first hydration shell radius of Martini). The residues that are in contact with PG form two groups. One group is located on the LOOP and the other on the globular part of N-domain (Fig. 5). For the first group of residues, the interaction with both lipid species occurs. They are located on the surface of the acceptor binding site and trace the way from the LOOP to the active site. Both PG and DAG are in tight contacts with the following residues T151, W182, D184, H185, W188, P189, F190, R195, S222 and F230. A snapshot of PG in the vicinity of catalytic H155 (the distance is 0.5 nm) is given in Fig. 6a. This configuration shows a possible scheme for DAG binding. In contrary to PG, DAG molecules did not approach to H155 closer than 1 nm during the trajectory. This may relate to the fact that in CG simulations, MGD1 was in its apo-form. We suppose that DAG binding will be facilitated in the presence of UDP-Gal, which will modify the topology of binding cleft (discussed in the following section). The other snapshot on Fig. 6b shows the PG leaned against W182, D184, H185, W188 and P189. It is worth noting that residue P189 is known to be essential for MGD1 activation by PG^16,17^. The adoption of this position by PG leaves some room for DAG to approach to H155. The following section provides the information on the AA refinement of protein with substrates and the activator PG.

The alternative binding site (second group of residues), located on the globular part of N-domain (Fig. 5), shows a high specificity for PG and is formed by residues remote from the LOOP and the active site. It is located on a helix of N-domain. The site is mainly formed by positively charged residues, R260, R263, S264, G266, L268, K269; they do not interact with DAG (Fig. 5).

### The model structure of MGD1 in complex with UDP-Gal, DAG and PG

**Figure 7:**
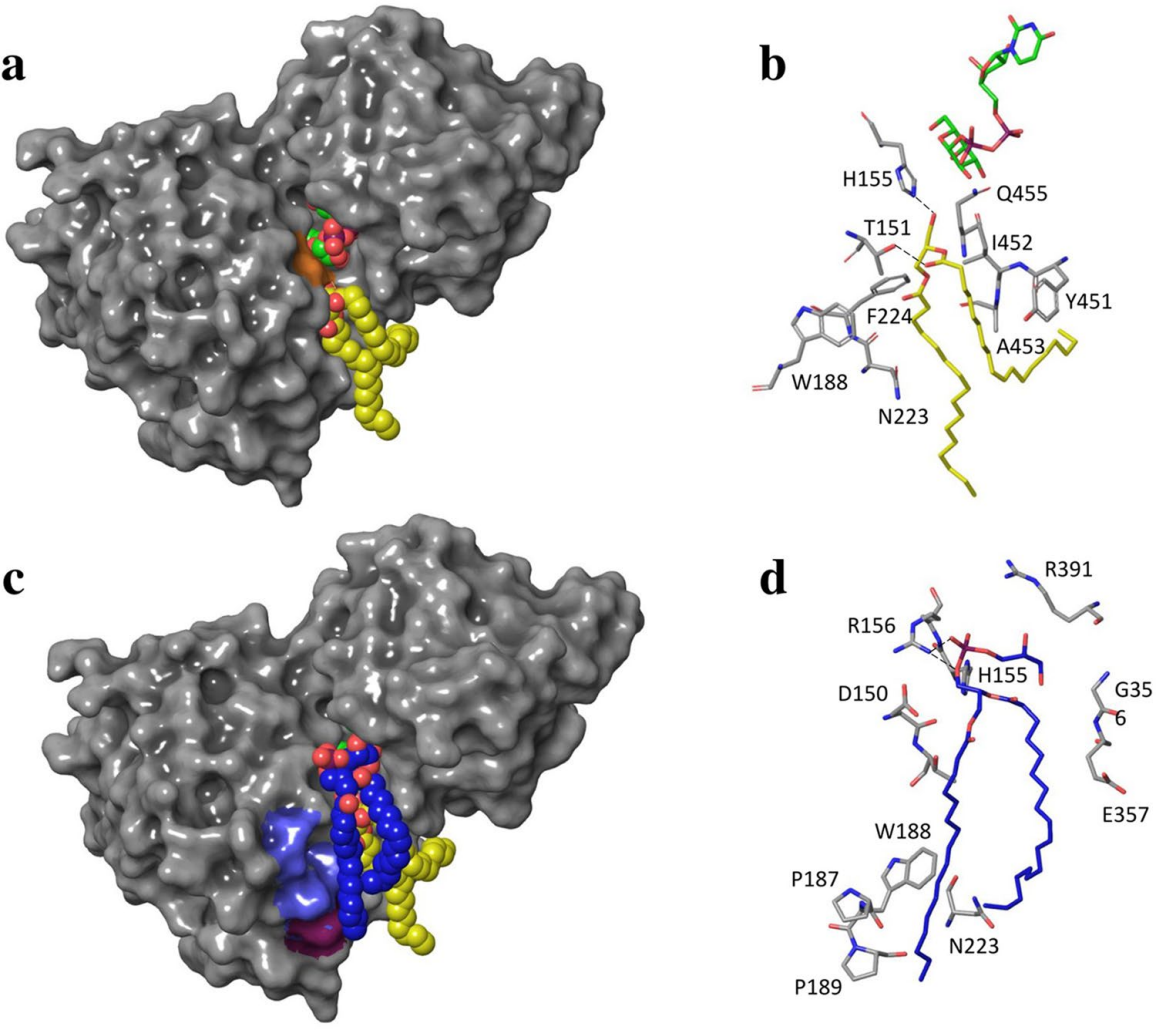
MGD1 in complex with UDP-Gal (carbon atoms in green) and DAG (carbon atoms in yellow) molecules (**a**, **b**), and PG (carbon atoms in blue) (**c**, **d**). The orange surface highlights catalytic H155; the ice-blue surface is for residues D184, H185, P187 and W188 and magenta—for P189.

Being inspired by the events when DAG and PG molecules are in close contact with the residues in the vicinity of the active site, we made a refinement of AA MGD1 complex with two substrates UDP-Gal and DAG and the activator PG molecule. Figure 7a,b illustrates the results of the molecular docking of DAG into MGD1 with bound UDP-Gal. DAG molecule forms two hydrogen bonds with H155 and T151; the rest of the interactions are with hydrophobic residues. A hydroxyl group of glycerol part of DAG is oriented towards the galactosyl moiety of UDP-Gal. The distance between O(H)…C1 is 0.6 nm which is expected to decrease during the sugar activation. The docking pose of PG in the complex of MGD1 with two substrates is given in Fig. 7c,d.

### The effect of membrane-binding on the concerted fluctuations of residues

To further illustrate the influence of membrane on MGD1, we studied the modulation of intrinsic protein dynamics induced by the lipid membrane by per residue fluctuation analysis. The simulations were carried out at the AA level to take the inter-protein hydrogen bond network into account explicitly. Since the CG model revealed the interactions of N-domain mainly with PG/DAG clusters, we, therefore, excluded glycolipids from the AA model. Furthermore, in the CG model, PG molecules interact with a large area on the surface of N-domain, including R260, R263, S264, G266, L268 and K269. Consequently, for the objective of the current section, we considered possible to use pure PG bilayer interacting with MGD1.

**Figure 8:**
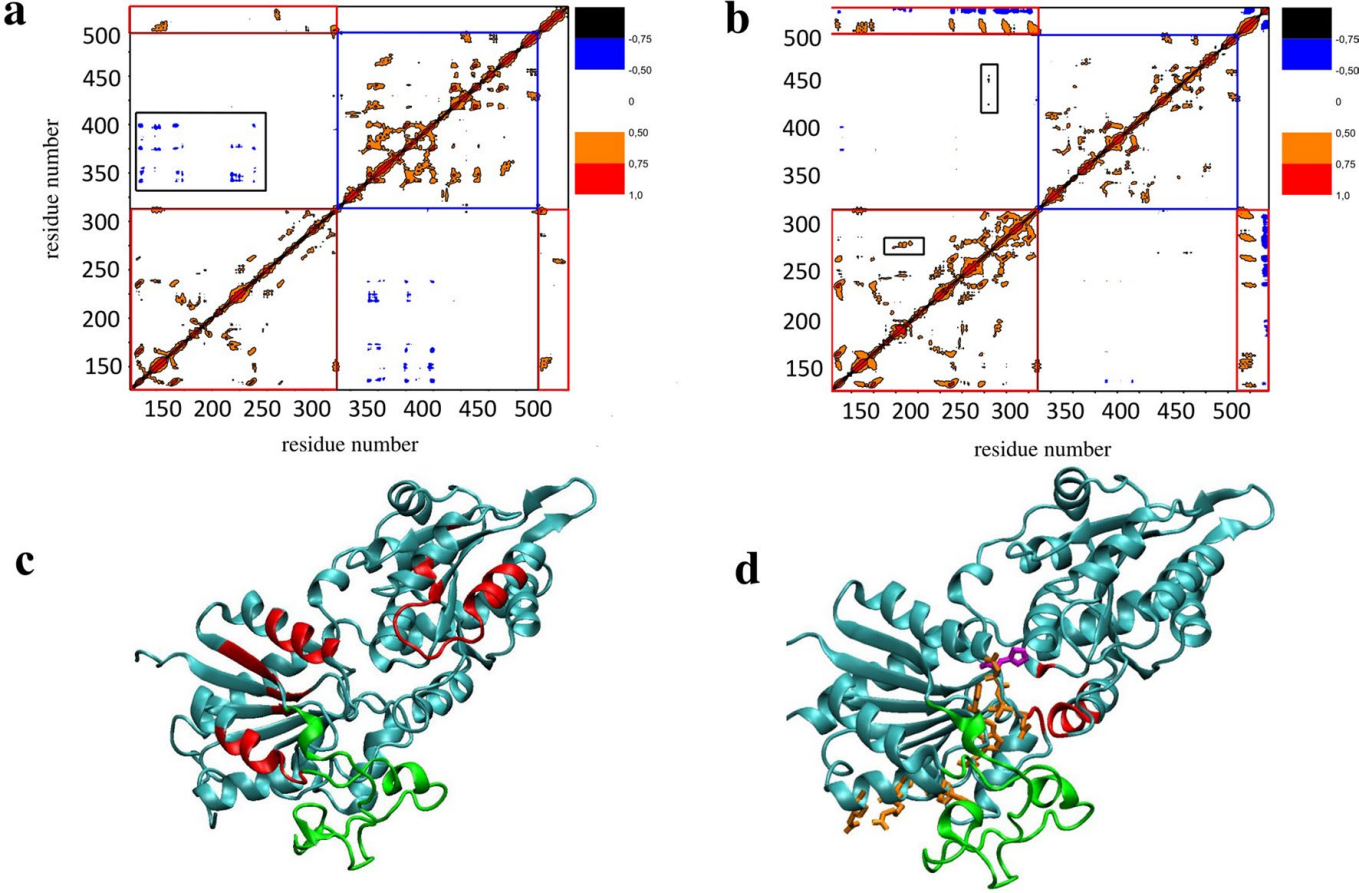
Cross-correlation maps for MGD1 in water (**a**) and MGD1 interacting with PG bilayer (**b**). The red and blue squares on maps indicate N- and C-domain, respectively. The groups of cross-peaks inherent only for free (**a**) and membrane-bound (**b**) protein state are framed in black. MGD1 spatial structure in water with residues showing inter-domain cross-peaks highlighted in red (**c**). MGD1 spatial structure upon interaction with PG membrane, residues leading to the appearance of new inter-domain cross-peaks are shown in red and those with concerted fluctuations in the N domains orange sticks (**d**). The catalytic H155 is in magenta. The LOOP is indicated in green.

The objective was to establish how the binding of MGD1 to the membrane surface modifies the dynamical network of the remote protein regions. To this end, the cross-correlation maps were calculated for MGD1 in water (Fig. 8a) and MGD1 attached to the membrane by N-domain (Fig. 8b). The maps represent the correlation coefficients for each pair of residues. The high value (absolute meaning from 0.5 to 1) indicates two residues with concerted fluctuations along the trajectory. For MGD1 in the water box, the cross-correlation maps revealed several cross-peaks corresponding to N- and C-domain residues correlated motions. As indicated in Fig. 8c, the residues with correlated motions cover a large area of surface residues in both domains. The interaction of MGD1 with the membrane led to more correlated fluctuations of residues in the N-domain. The pattern of cross-peaks significantly changed. First, the inter-domain cross-peaks became simpler, the group of peaks framed in Fig. 8a disappeared, and new ones appeared, one between residues belonging to different domains (N- and C-domains) and the other to those placed in the same N-domain. The new group of inter-domain cross-peaks corresponds to the area where the DAG binding site is located (Fig. 8d). When protein interacts with the membrane, there is an increase in the number of cross-peaks of the N-domain as compared to protein in water. From the tracing of new cross-peaks, one may extract the following sequence of residues. They form the inter-connected concerted network: L259, R263, **H256,** H289, **W287,** H251, **D279,** D150 and T151. Some residues (those indicated in bold) along this path were proved to be essential for catalysis^15^.

## Discussion

Whereas many essential questions about the mechanism underlying the catalytic activity of MGD1 had been answered through experiments^17,28^, a central question remained on how MGD1 recognizes and interacts with DAG. Previous data suggested a role of the LOOP in the capture of DAG. Indeed, the deletion of part of this region had a significant impact on the rate of binding to DAG^15^. Unfortunately, X-ray diffraction studies could not provide any structural insight into the LOOP, presumably because of enhanced conformational flexibility. The results of our in silico analysis confirmed the disordered conformation of the MGD1 LOOP, in the absence of stabilization by the membrane. The current work describes the molecular mechanism of DAG capture by MGD1 with LOOP assistance. Albeit not being an ideal situation, the present challenge of giving an inside description of the enzymatic mechanism is not unique. A somehow similar case was reported for the glycosyltransferase that synthesizes glycoglycerolipids in *Mycoplasma genitalium* membranes^29^. The authors invoke the role played by an amphiphilic peptide undergoing helix formation and its subsequent influence on the catalysis.

MGD1 needs anionic lipids such as PA, PG or SQDG to be active^16,17,30^. Enzyme regulation by anionic lipids has been demonstrated in other membrane-associated glycolipid synthases, such as those from *Mycoplasma genitalium*^31^ and *Acholeplasma laidlawii*^32^. Rocha et al.^15^ hypothesized that PG was located next to the DAG binding site and helped MGD1 to bind the DAG molecule. Using membrane models that mimic the natural environment of MGD1 bound to IEM (consisting in a mixture of MGDG/DGDG/PG/DAG), we evidenced a mechanism whereby PG molecules generate nano-sized clusters that embed DAG molecules. Such a cluster formation has a double advantage. The first advantage is that the DAG acceptor substrate and PG activator come spatially close to each other. Thus, they can be recognized by the protein together. Second, the accumulation of DAG in PG clusters increases the local concentration of DAG, which amounts to less than 1 percent of overall lipid content in the membrane of the chloroplast. The size of the clusters, estimated from simulations, is large enough to accommodate the N-terminal domain of MGD1. Throughout its interaction with the membrane, MGD1 may benefit from an additional accumulation of DAG due to favourable contacts with mainly neutral and aromatic LOOP residues. Such an additional clustering effect of DAG upon MGD1 binding was also observed using a DAG-PG (1:3) membrane model^17^.

Results from previously reported mutagenesis experiments established that some residues are crucial for MGD1 activity^15,16,33^. Some of these residues, including W188 and P189, belong to the LOOP. The results of the CG MD simulations, provide a direct vision of these interactions. These residues establish tight contacts with PG and DAG molecules and direct them into the active site. Intriguingly, in the course of their trajectories, PG develops interactions with the catalytic residue H155 and neighbouring R156.

Furthermore, we extended the model of the active complex throughout an AA representation. To this end, the other substrate UDP-Gal was added first to MGD1. The results of the docking procedure revealed that the DAG molecule could be accommodated in the cleft between N- and C-domains. The interaction occurs through hydrophobic contacts and the establishment of two hydrogen bonds with T151 and H155. The PG molecule interacts with both DAG and protein residues that trace the way from the LOOP to the active site. In this complex, the PG polar head interacts with R156 throughout its negatively charged phosphate moiety and approaches to H155 (both distances are about 0.6 nm). This location is ideally suited to create an acid–base charge relay system, in the form of a PG-His catalytic dyad, as it was recently proposed for MGD1^17^. Such a catalytic dyad, analogous to the more classical acid–base dyad (i.e. Asp-His or Glu-His) observed in some bacterial glycosyltransferases^34^, should facilitate the deprotonation of the nucleophile OH group of DAG acceptor by H155. Altogether, our results suggest that the observed lipid reorganization with the formation of PG-DAG clusters create an optimal platform for MGDG synthesis, a prerequisite for coping the massive demand of galactolipids upon light illumination of plastids. Therefore, PG plays a dual role: it ensures a very rapid capture of DAG by MGD1 and helps the enzyme to perform the catalytic reaction. This hypothesis is reinforced by the observation that MGD1 showed the fastest kinetics of binding on a DAG-PG monolayer^18^. SQDG, the other major anionic lipid in chloroplast membranes, can probably fulfil the same role as PG. Recent data demonstrated that SQDG could also activate MGD1 by a mechanism similar to PG^17^. It must be stressed that PG and SQDG are functionally redundant in planta^35^ and that the total anionic lipid content (~ 15%) is probably the most critical parameter rather than the respective levels of PG and SQDG^36^. It would be interesting to see if SQDG can induce a similar DAG clustering effect as PG.

In the present work, we also considered the influence of the anionic PG on the modulation of intrinsic protein dynamics. The analysis of MD trajectories revealed a group of basic residues of the N-domain that formed an isolated spot making tight interactions with PG. Further, we extended the analysis to study the influence of PG binding to this area on the intramolecular protein dynamics. The study revealed an increase in the concerted fluctuations of the residues belonging to the N-domain. Such an increase presumably results from their interactions with the membrane. The changes in the dynamics of residues L259–R263 propagate to the residue that forms the hydrogen bond with DAG and orients the substrate in the binding site. The perturbations affect the following residues: H256, H289, W287, H251, D279, D150 and T151. Residues H256 and D279 were experimentally proven as critical for MGD1 activity. When mutated to alanine, they demonstrated less than 5% of the wild type enzyme activity^15^. Residue W287 was previously considered as a residue potentially interacting with PG^16^. However, the mutation W287 into alanine yielded a mutant protein quite unstable upon purification, and we were unable to detect any activity either with PG or PA (data not shown). However, from the present work, it hardly can be involved in direct interactions with DAG or PG, because its accessible surface area amounts to zero. One may suggest that replacing its massive side chain with a smaller one probably perturbs the protein folding in this region. Nevertheless, the analysis of correlated motions revealed that its massive side chain is instead responsible for tight contacts inside the protein globula to translate the dynamic perturbations across the N-domain from the membrane surface to the active site.

The modulation of intrinsic protein dynamics observed upon MGD1 membrane binding may also be indicative of a cooperative effect and thus of a form of allosteric regulation that will be driven by changes in protein motions^37^. Such an observation may be particularly relevant to explain the allosteric behaviour previously described for PA^16^. PA, which is a precursor for DAG, is barely detectable in chloroplast membranes. It is believed to play an essential role in the regulation of phospholipids and galactolipid syntheses in plants^38^. The mechanism of MGD1 activation by PA is still puzzling. PA and PG seem to proceed through different mechanisms with a synergistic effect suggesting distinct binding sites^16^. Although there is currently no indication on its potential binding site on MGD1, one can hypothesize that, at least in vitro, PA binds to the active site in a different way compared to PG, while maintaining H-bonding with the catalytic His residue. The possibility of a second regulatory binding site for PA could also be considered to explain its allosteric behaviour measured in vitro. Such a regulatory site could play a major role in controlling MGD1 activity in the chloroplast. It would be interesting to show how PA influences the MGD1 dynamics. The elucidation of the mode of action of a monotopic enzyme sitting at the surface of the IEM required the assistance of multiscale computer modelling. On the one hand, the extensive spatial and temporal scale processes resulting from the membrane reorganization and the dynamical nano-scale cluster formation required the use of CG based methods.

On the other hand, the modelling of the establishment of the protein–lipid substrate/activator complex required a description at the atomic scale level. In the CG representation of such a complex system, the grouping of four heavy atoms decreases the number of degrees of freedom. It smoothes the landscape of the potential energy surface and facilitates the occurrence of the conformational transitions and the analysis of the structures belonging to these low energy minima. Both bonded and non-bonded features are structurally related to those in the AA representation. Thus, the evolution of the molecular system in the course of the CG MD trajectory can be achieved without sacrificing of atomistic details of the system. Every frame of CG MD trajectory can be back mapped to the AA representation using CG beads as a scaffold. However, the critical limitation of the method originates from the absence of hydrogen bonds and the subsequent lack of information about the intermolecular distances and the spatial orientation. For lipid dynamics, which are regulated mainly by hydrophobic interactions, one may neglect the hydrogen bonding.

On the contrary, a proper description of hydrogen bonds is essential to understand the formation of the elements of secondary structures. Protein hydrogen bonds significantly contribute to both the large-scale conformational motions and low scale residue fluctuations in the protein. Also, the formation of hydrogen bonds drives the recognition of the protein and its substrate partially. To consider the crucial importance of hydrogen bonds, the refined AA structure of the protein–ligand (lipid-substrate and lipid-activator) complex is required, as extracted from the protein–ligand contact map in CG MD simulations. The hydrogen-bonding network is expected to be also essential for intra-molecular dynamics. We extracted the concerted fluctuations over the AA MD trajectory for each pair of residues and compared these values for the protein in water and the protein bound to the lipid membrane. The AA MD simulation can decipher the mechanics of protein fluctuations and to evaluate the induced action of ligands binding on protein conformational dynamics. It is, however, a meaninglessly long process to simulate, at this level, such a complex system as the IEM of the size sufficient to be representative for the interactions with protein, MGD1.

### Methods

### Lipid bilayer composition

Four bilayer models (listed in Table 1) were considered with the major lipids known to compose the chloroplast membrane, along with the different levels of their content. For simplicity, SQDG was excluded from the model membranes. Indeed, this sulfolipid was shown to be not essential under normal plant growth conditions and can be substituted by PG^35,36^. The MGDG and DGDG were considered to have the majority of polyunsaturated fatty acid tails^13^. The tails of PG and DAG were mostly unsaturated^30^.

Two sizes of membranes were constructed for each of these models having a selected glycolipid composition. The small systems contained 200 lipids, i.e., 100 lipids per leaflet, and had an initial size of box 8 × 8 × 8 nm. The choice of such dimensions does not lead to membrane undulation. Besides, it provides a direct comparison between the structural parameters (area per lipid, area compressibility moduli, membrane thickness) and the results of simulations of thylakoid membrane calculated at both CG and AA levels of molecular representation published by van Eerden et al.^19^. The larger systems having the initial size of 20 × 20 × 8 nm were used to study the dynamical characteristics of the lipids and to study the membrane–MGD1 interactions further.

### Protein modelling: loop construction and insertion to the membrane

The primary sequence of MGD1 starts with a chloroplast transit peptide in N-terminal position (amino acids 1–106) followed by a short sequence (amino acids 107–136) predicted to be disordered and not necessary for the activity of the enzyme^16^ and by the catalytic domain that comprises the amino acids 137 up to 533.

Two crystal structures of the catalytic domain of MGD1 are currently available, in its apo-form (PDB code: 4wyi at resolution 2.5 Å), and in complex with UDP (PDB code: 4x1t, 2.25 Å)^15^. One of the peculiarities put forward by the resolution of the two structures is the lack of electron density corresponding to a region in the N-domain.

The LOOP (residue number from 182 to 230) of MGD1 was constructed de novo using i-TASSER, the unified platform for automated protein structure and function prediction^39^. The spatial coordinates of residues adjacent to the first and the last residues of the LOOP, as taken from the crystal structure, were used as positional constraints. Five most energetically favourable models resulted from the construction; they were kept for further inspection.

Following the reconstruction of the LOOP, the complete protein was pre-oriented to the bilayer using the Positioning of Proteins in Membrane (PPM) service, which minimizes the energy for transferring the hydrated protein to the membrane^27^.

### Coarse-grained and all atoms simulations

The computational task was performed on (1) MGD1 in water, (2) lipid membrane in water and (3) MGD1-lipid membrane in water. All model systems were built up with INSANE (INSert membrANE)^40^ and CHARMM-GUI building tools^41,42^.

**Figure 9:**
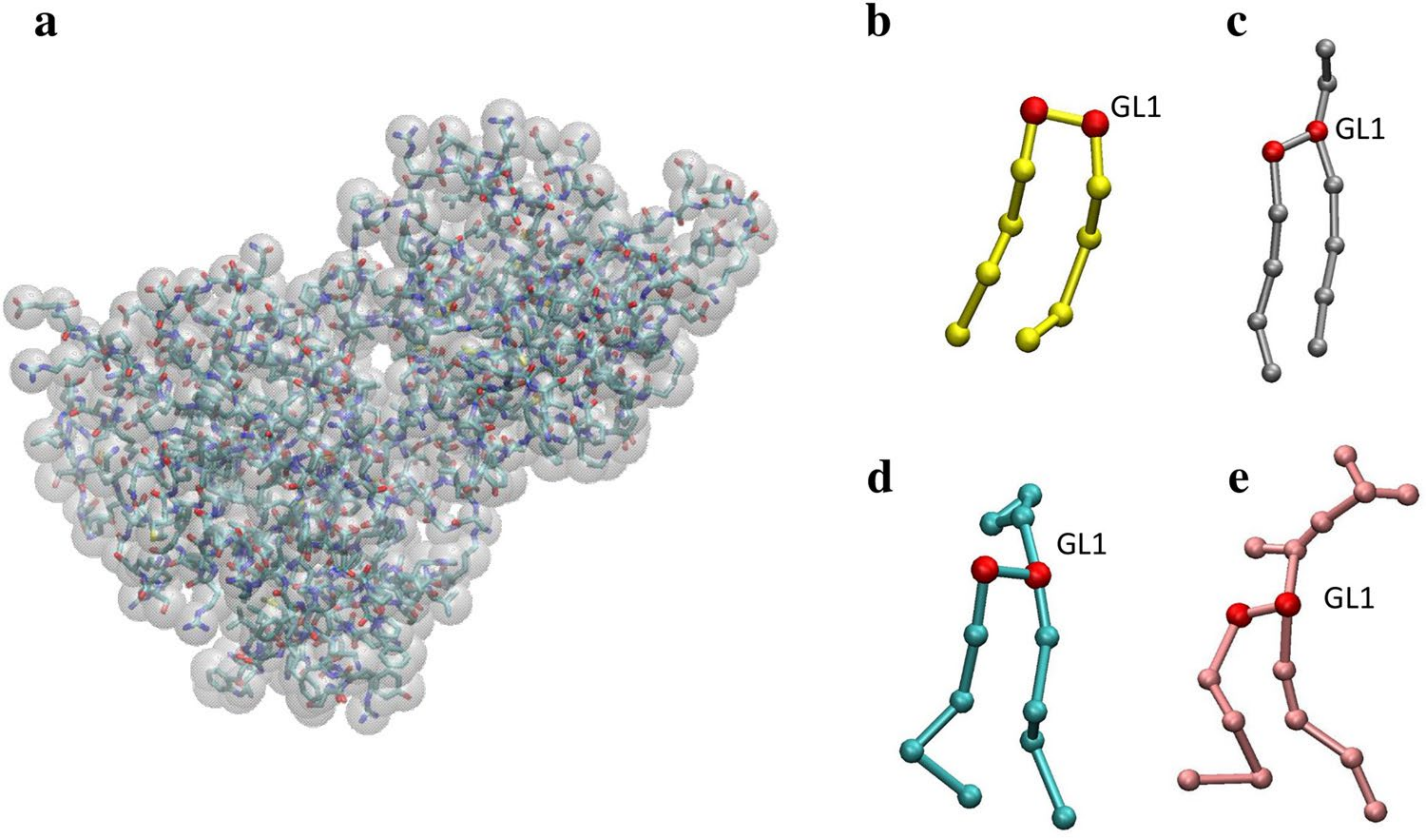
Martini CG representation of MGD1 (**a**), DAG (**b**), PG (**c**), MGDG (**d**) and DGDG (**e**). For the protein, the CG and AA representations are superimposed for clarity. In the scheme of lipids, the beads of glycerol part are highlighted in red, the bead of glycerol group GL1, used for the analysis of lipid behaviour in the membrane, is indicated.

The Martini force field v2.0^43,44^ was used to model the intermolecular interactions. Figure 9 illustrates the Martini CG representations of MGD1 and lipids of interest for the present work.

The following systems were simulated:

1. MGD1 in water with elastic network model (ElNeDyn)^45^, Go-Martini^46^, and without any restraints—each of 10 μs in the production phase.
2. Four bilayers M1 to M4 without any restraints—each of 6–10 μs in the production phase, except for M2. For the latter, phase transition occurred as a result of the occurrence of large undulations. Therefore, six trajectories of 1 µs were accumulated for M2 to reach reproducible results of the clustering of lipids.
3. MGD1-glycolipid membrane with ElNeDyn restraints on protein, which was repeated without any restraints, over a production period of 10 μs.

All MD simulations were performed with the GROMACS 5 package^47^ with the standard parameter settings for the Martini force field. All the systems were equilibrated with counter ions Na^+^Cl^−^ to reach the overall amount of 150 mM. The energy was minimized with the steepest descent algorithm. Then, four equilibration stages followed, where the position restraints on the headgroups were gradually decreased from 200 to 0 kJ mol^−1^ nm^−2^, and the time step for leap-frog integrator was increased from 2 to 10 fs. The production phase was performed with a 10 fs time step according to that used in Ref.^19^. The systems were calculated in the isothermal-isobaric (NpT) ensemble. The temperature was kept constant at 293 K using the velocity rescale coupling algorithm with 1 ps time-constant. Lipids, solvent, and protein (if any) were coupled separately. The pressure was kept constant at 1 bar with a coupling constant 5 ps and compressibility of 3 × 10^–4^ bar^-1^ using Berendsen barostat. Electrostatic interactions were calculated using reaction-field with cut-off 1.2 nm and dielectric constant of 15. Van der Waals interactions were also cut-off at 1.2 nm using a potential-shift-Verlet modifier. Three dimensional periodic boundary conditions were used for the simulation box.

The CHARMM36 force field was used for the series of AA simulations^48^ to investigate the following cases: MGD1 in water over a 600 ns length of the production phase and MGD1 in PG bilayer, over a 200 ns length of the production phase.

For AA simulations, the MGD1 protein was placed on the PG bilayer and counter-balanced by Na^+^ and Cl^−^ ions to reach a total concentration of 150 mM. The systems were calculated in the isothermal-isobaric (NpT) ensemble. The temperature was kept constant at 300 K using the velocity rescale coupling algorithm with 1 ps time constant. Lipids, solvent, and MGD1 were coupled separately. The pressure was kept constant at 1 bar with a coupling constant 5 ps and compressibility of 4.5 × 10^–5^ bar^−1^ using Parinello–Rahman barostat. Electrostatic interactions were computed using PME with cut-off 1.2 nm. Van der Waals interactions were also cut-off at 1.2 nm using the potential-shift-Verlet modifier.

### Data processing and analysis

The analytical tools implemented in GROMACS provide a way to the structural description of the membranes. The surface area per lipid (SA) was calculated by dividing the area of the membrane surface by the number of lipids. The compressibility modulus (K_A_) was as described in Ref.^49^,

$${K}_{A}= \frac{kT{A}_{0}}{N\langle {\left(A-{A}_{0}\right)}^{2}\rangle },$$

where A is the surface area per lipid (SA), A_0_ is the average surface area per lipid, N is the number of lipids per leaflet, T is the system temperature. The membrane thickness was determined as the distance between the maxima of the density of lipids atoms along the normal to the membrane plane.

To characterize the dynamical properties of the lipids, the lateral diffusion coefficient was calculated from the mean square displacement (MSD) of the molecules in the membrane plane using the GROMACS gmx msd tool. The MSD = <|r(t + t0) − r(t0)|^2^ > where r corresponds to the position of the molecular center-of-mass, angular brackets denote time and ensemble averaging. The lateral diffusion coefficient (D) was calculated as a slope using Einstein relation y = 4Dt + c.

The GROMACS gmx mindist tool provides a way to calculate the normalized amount of contacts between lipid species. The lipids are in contact if their glycerol group GL1 beads appear within the first solvation shell of the other lipid beads, i.e., at the distance 0.8 nm or less. The lipids of the one type were added to the same cluster if GL1 beads were within 0.8 nm. The number of lipids in the resulted clusters was calculated using GROMACS gmx clustersize and cluster tool, correspondingly. Flip-flopping of lipids between the leaflets was counted as a number of membrane-crossing-events along the z-axis. The z coordinate as a function of time was calculated using the VMD program^50^.

Normal Mode Analysis (NMA) of Cα-based anisotropic network model was applied to the protein molecule for prediction of slow internal motions using ANM Web Server 2.1^26^.

Principal Component Analysis (PCA) was performed using the tools implemented in Gromacs package (gmx covar and gmx anaeig). The projections of a trajectory on the eigenvectors of its covariance matrix are called principal components (PC’s). The motions of Cα atoms along the first two principal components were calculated.

Cross-correlation matrix shows the correlation for each pair of residues, centred on Cα atoms. The translational and rotational motions of the protein as overall were previously removed before the calculation of cross-correlation matrix.

The elements of cross-correlation matrix for i and j atoms were calculated as follows:

$$Cij=\frac{[\Delta {\overrightarrow{r}}_{i}(t)\Delta {\overrightarrow{r}}_{j}(t)]}{{\left(\left[(\Delta {\overrightarrow{r}}_{i}{(t))}^{2}\right]\left[(\Delta {\overrightarrow{r}}_{j}{(t))}^{2}\right]\right)}^{1/2}},$$

where $\Delta {\overrightarrow{r}}_{i}\left(t\right)={\overrightarrow{r}}_{i}\left(t\right)-\left[{\overrightarrow{r}}_{i}\left(t\right)\right]$ determining the displacement of atom i from its MD-averaged position. The angular brackets denote an MD-averaged value.

### Docking of UDP-Gal, PG and DAG to MGD1

The following docking procedure of UDP-Gal and DAG to MGD1 molecule was performed using HADDOCK2.2 webserver^51^. First, the UDP-Gal was docked to the unliganded MGD1. The correct positioning of UDP moiety was controlled by the knowledge from the crystal structure of MGD1 with UDP bound (PDB code: 4x1t). The knowledge about the position of sugar moiety was taken from the crystal structures of homologous enzymes, i.e., the red grape enzyme UDP-glucose: flavonoid 3-*O*-glycosyltransferase (GT1 from *Vitus Vinifera*, PDB code: 2c1z, and UGT71G1 from *MedicagoTruncatula*, PDB code: 2acw). Afterwards, the DAG and PG molecules were consequently docked to the MGD1-UDP-Gal complex. The interaction of DAG with the catalytic residue H155 was used as a constraint.

## Supplementary information

Supplementary Video 1.

Supplementary Video 2.

Supplementary Information.
