# Supplementary material for: Unraveling the complex enzymatic machinery making a key galactolipid in chloroplast membrane: a multiscale computer simulation
Source: Sci Rep. 2020 Aug 11;10:13514. doi: 10.1038/s41598-020-70425-z (PMC7419546; doi:10.1038/s41598-020-70425-z)
Supplement: Supplementary file 3 — Supplementary Information. [file 41598_2020_70425_MOESM3_ESM.pdf]

UNRAVELING THE COMPLEX ENZYMATIC MACHINERY MAKING A KEY GALACTOLIPID IN CHLOROPLAST MEMBRANE: A MULTISCALE COMPUTER SIMULATION

Olga Makshakova, Christelle Breton and Serge Perez

Supplemental Information

S1. Membrane characterization

Table S1.1. Contact percentage between monogalactosyldiacylglycerol (MGDG) and digalactosyldiacylglycerol (DGDG), phosphatidylglycerol (PG) and diacylglycerol (DAG) in inner envelope membrane (IEM) models M1-M4.

	**MGDG**	**DGDG**	**PG**	**DAG**	**Expected values from ideal mixing**
**M1**					
PG			67	80	75
DAG			33	20	25
**M2**					
MGDG	59	67	19	21	50
DGDG	33	23	13	16	30
PG	3	4	28	36	10
DAG	4	6	40	27	10
**M3**					
MGDG	56	63	44	27	50
DGDG	31	23	22	12	30
PG	10	11	23	26	15
DAG	3	2	11	35	5
**M4**					
MGDG	49	55	26	16	40
DGDG	35	24	22	16	30
PG	13	17	38	56	25
DAG	2	3	14	12	5

a

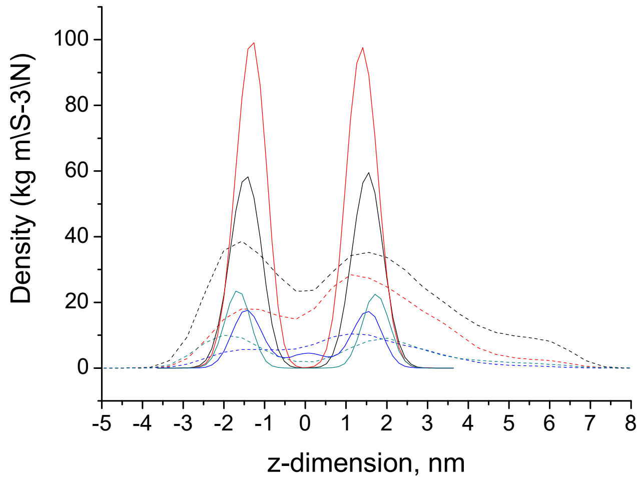

b

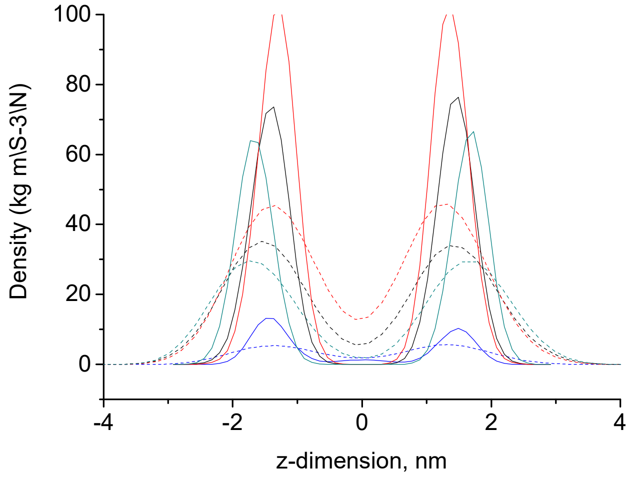

Figure S1.1. The density profiles for GL1 beads along z-coordinate in the small (solid lines) and the large (dashed lines) variations of M2 (a) and M4 (b) model membranes. Color coding: red for MGDG, black for DGDG, green for PG and blue for DAG.

a

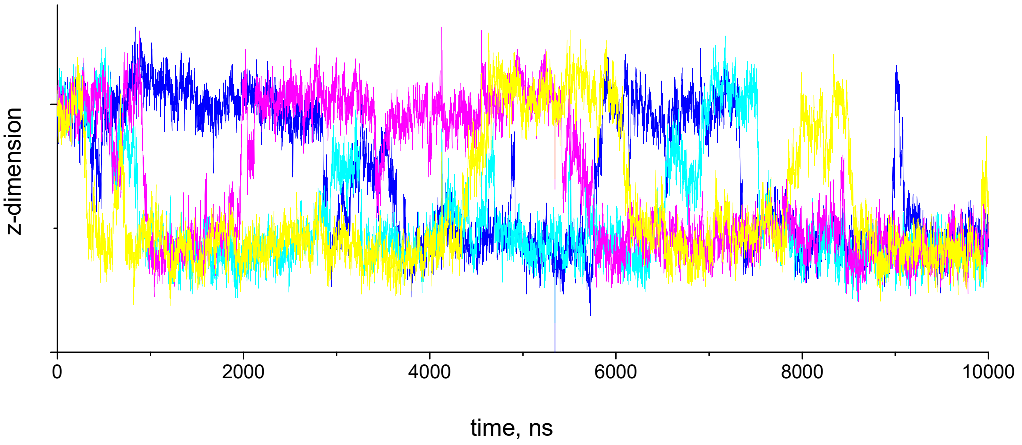

b

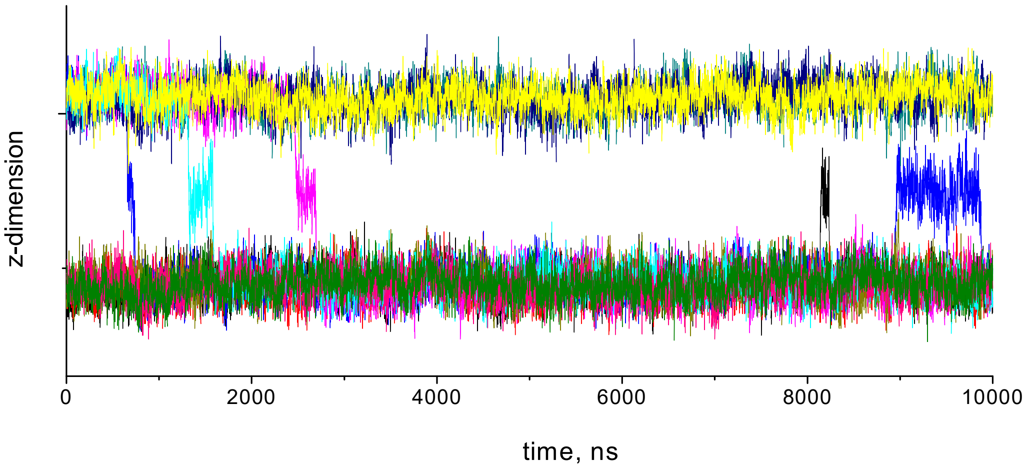

Figure S1.2. The Z-position of GL1 bead of DAG molecules in M2 (a) and M1 (b) model membranes.

2. Loop reconstruction

Two sets of coordinates for MGD1 are available in PDB for the protein apo-form 4wyi and for holo-form with UDP bound 4x1t. For apo-form, the electron density for amino acid sequences: 139-181 and 231-523, could be localized and their 3D structures refined. As for the holo-form, the coordinates for amino acids: 142-179 and 243-525 are present in the PDB file. The following procedure was used to establish a 3D structure model for full protein.

We used the *de novo* modelling algorithms implemented in i-Tasser and, starting with the coordinates of the MGD1 part of apo-form (PDB code: 4wyi). Then, we constrained the positions of first and last residues of the LOOP. It resulted in five most energetically favourable models.

Among the five models, three revealed structures, with rmsd values for Cα-atoms varying from 0.5 to 1.1 Å. They were considered as one model (referred to as Model1, Figure S2.1). Two models had rmsd values larger than 6 Å (Model1 to Model4 7.9 Å, Model1 to Model5 6.2 Å, Model4 to Model5 6.5 Å). Being so distinguishable, they were considered as different models.

Model 1 is the most compact; the largest part of the LOOP interacts with the N-domain and blocks the UDP-Gal banding site. In Model4 and Model5, the bulky moiety of the LOOP lies away from the UDP-binding site. The difference between these two models lies in the orientation of the LOOP with respect to the core of the protein (see Fig. S2.1).

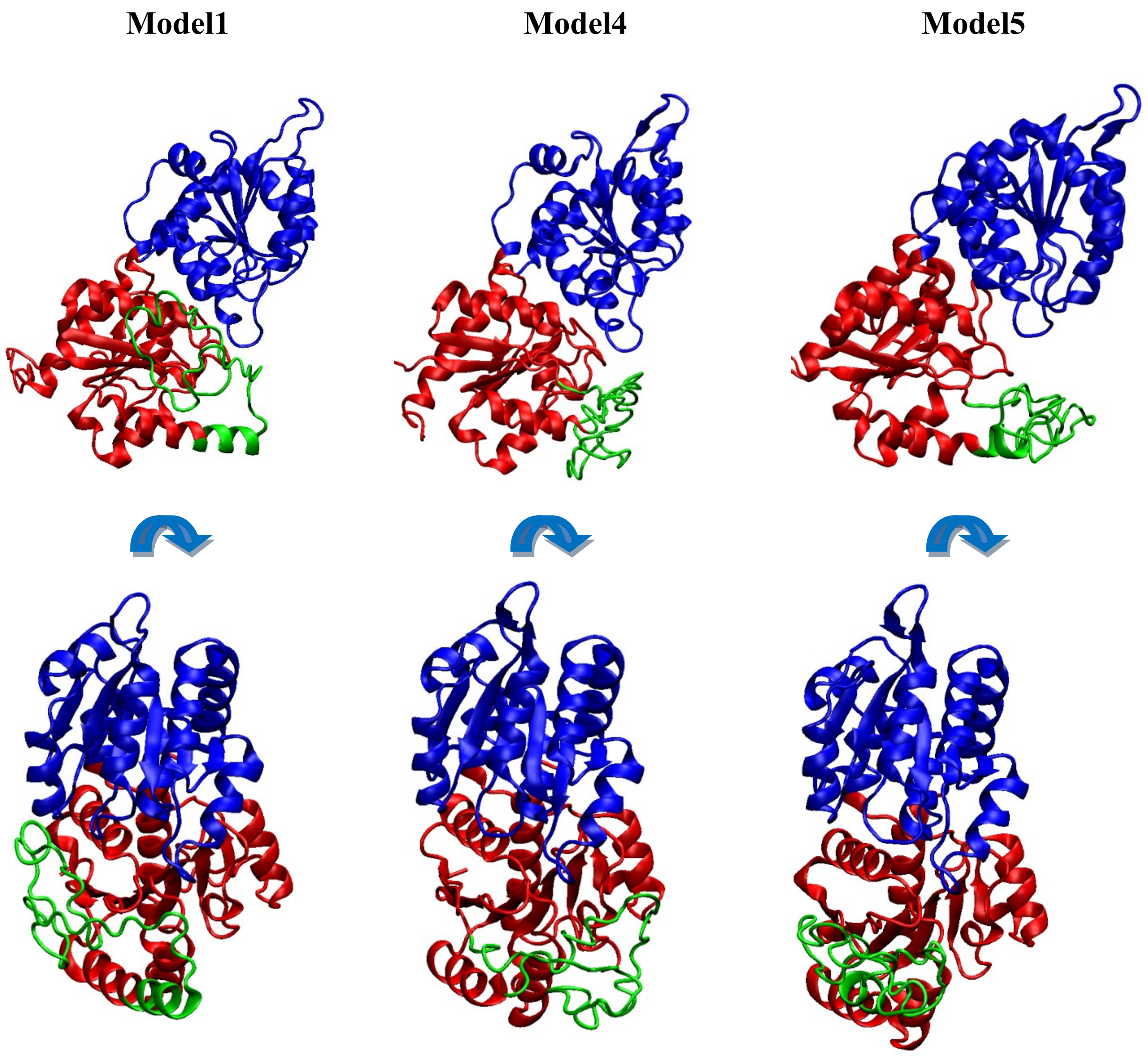

Figure S2.1. Three full sequence models of MGD1 molecule, obtained on the base of X-ray structure (pdb code: 4wyi) and *de novo* loop reconstruction. Color coding is following C-domain – blue, N-domain – red and the reconstructed loop within N-domain – green.

S3. Protein motions (out of membrane)

Anisotropic Network Model

The Normal Mode Analysis (NMA) with Anisotropic Network Model (ANM) [1] was performed to characterize slow motions in MGD1. NMA analysis was performed for all three models, using http://anm.csb.pitt.edu/. In ANM, the nodes are centred on the Cα atoms; the overall potential is given as the sum of harmonic potentials between the interacting nodes. Information about the orientation of each interaction with respect to the global coordinates system is considered within the Force constant matrix (i.e. Hessian matrix) and allows prediction of anisotropic motions.

The calculation of the B-factors revealed that the most substantial flexibility occurs in the 182-230 sequence; this is in accordance with the experimental data. The calculated B-factors for Model4 and Model5 are in close agreement. As for Model1, the LOOP has the lowest flexibility among the three models; because of the large number of contacts with the globular part of the protein.

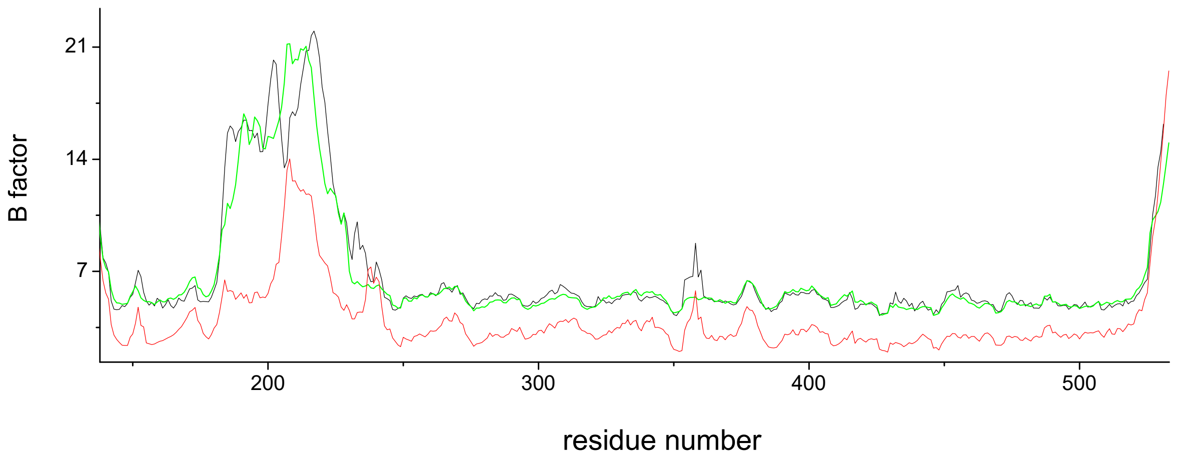

Figure S3.1. B-factors calculated in the framework of Anisotropic Network Model. Color coding: red – Model1, black – Model4 and green – Model5.

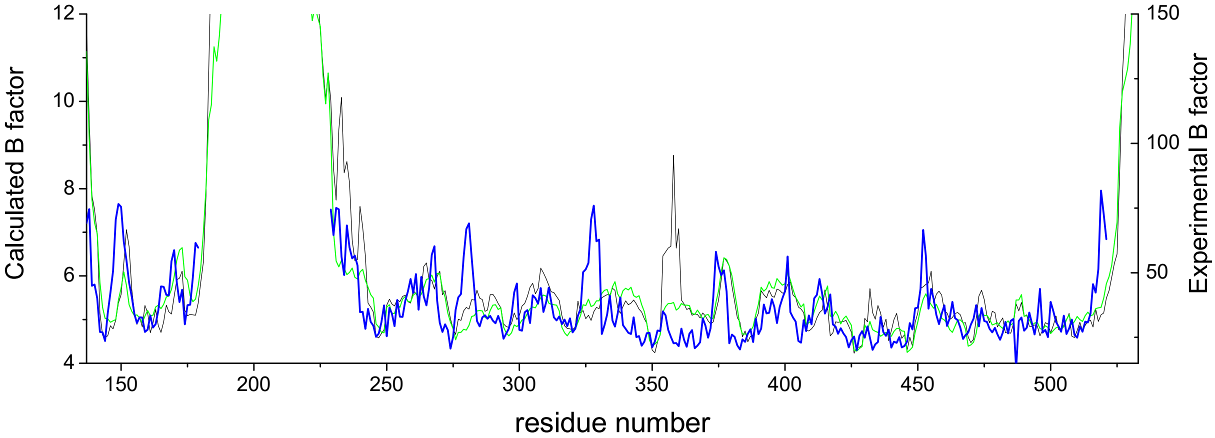

Figure S3.2. Experimental (blue) and calculated (black – Model4 and green – Model5) B-factor values.

The collective motions of MGD1 calculated in the framework of ANM can be described as mutual twisting and approaching of N and C-domains (two first normal modes are shown in Fig. S3.3).

Mode1 **Mode2**

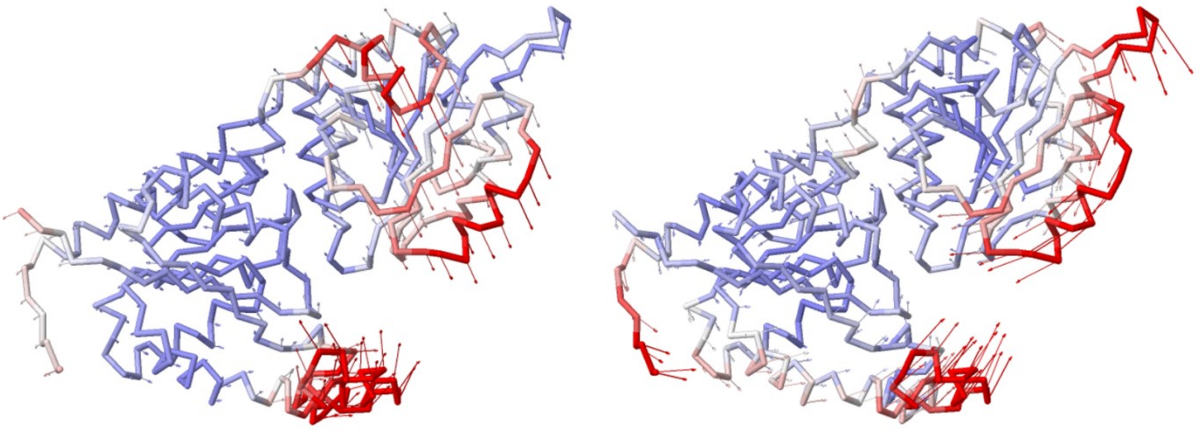

Figure S3.3. Visualization of the slow modes calculated using ANM. The colors and vector lengths are in according with the self-fluctuations, red indicates large fluctuations, and blue indicates small fluctuations.

Conformational dynamics of MGD1 in water box, calculated at the AA and CG levels of representation.

All-Atom simulations

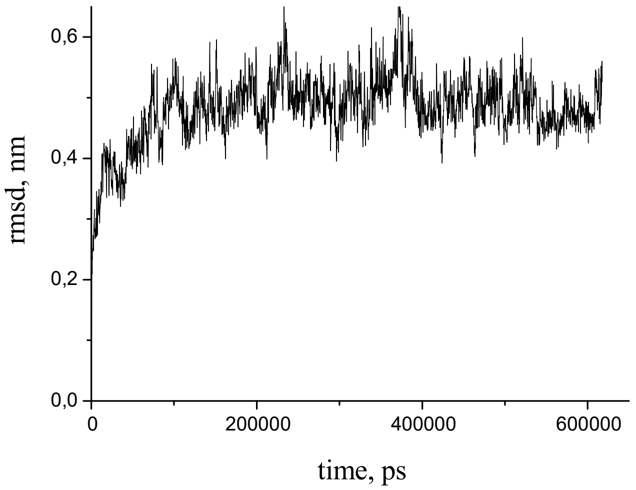

Figure S3.4. Rmsd values for Cα-atoms of MGD1 in water box calculated along AA MD trajectory.

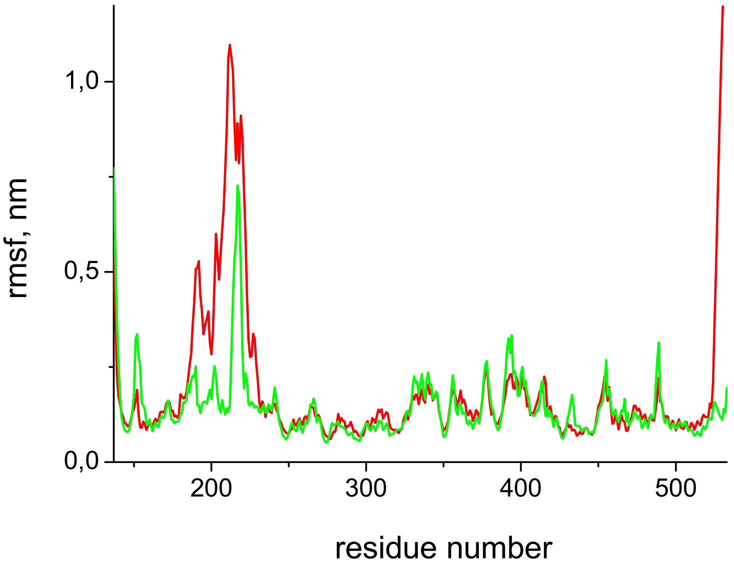

Figure S3.5. Rmsf values calculated along AA MD trajectory of full sequence Model4 (green) and Model5 (red) of MGD1 in water box.

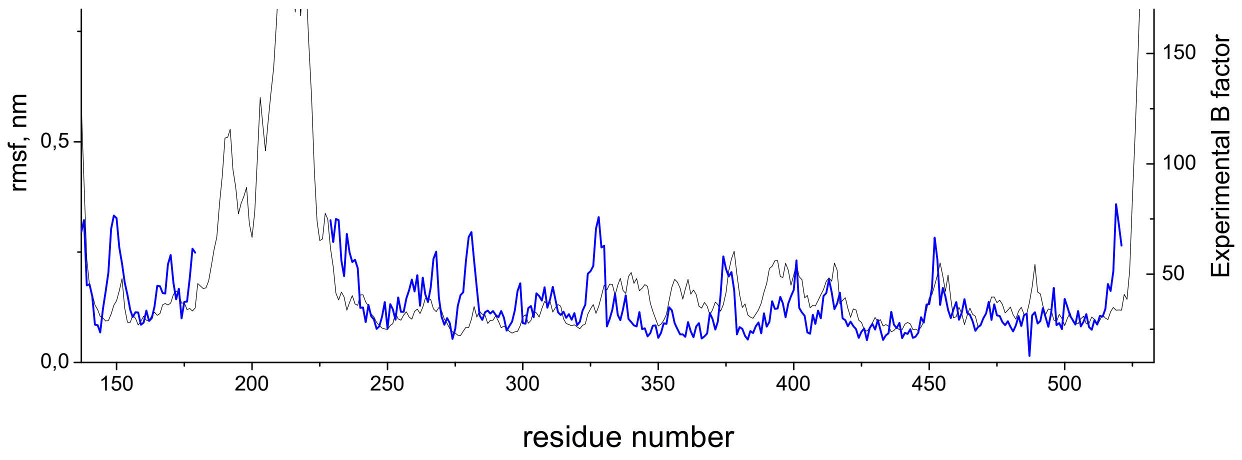

Figure S3.6. Rmsf values calculated along AA MD trajectory of MGD1 in the water box and experimental B-factor values (blue).

Coarse-Grained simulations

Comparison of the application of different Martini [2] models with varying types of approximation on the protein residue fluctuations: elastic network model (ElNeDyn) [3], Go-Martini [4] and without any constrains.

ElNeDyn incorporates additionally harmonic bonds and is widely used to preserve the native structure of the protein. Unbreakable harmonic bonds hinder the study of unfolding and folding processes, seemingly including the large-scale loop motions. Gō-like models use Lennard–Jones interactions based on the contact map of the native protein structure instead of the harmonic bonds. They are consistent with advanced-sampling methods (i.e. metadynamics). The Martini method applied to a protein without constraints may lead to non-physical protein dynamics. Nevertheless, it is documented that both Martini and Go could recapture the dynamics of a model protein, Eglin C, to a significant extent, as indicated by root-mean-square fluctuations and correlated motions [5].

Given that MGD1 is a bilobal protein, the skewing and twisting in the region of a short linker are essential for intrinsic protein dynamics, to which the LOOP significantly contributes. In a result of these collective motions, two domains shift in respect to each other. The first two normal modes calculated in the framework of ANM model are illustrated in Figure S3.3. The PCA analysis allowed extracting the most collective motions along the trajectory, which are in close agreement with those predicted in the framework of ANM. Altogether, the agreement with experimental B-factor and slow collective motions calculated by ANM validates the Molecular Dynamics simulation under the selected conditions.

Thus, the simulations of protein in water box support high flexibility of the LOOP, but hardly provide a cause to prefer one of the extreme positions of the LOOP for further analysis. This is why the interactions with membrane should be taken into account to predict the LOOP

conformation in protein functional (active) state, (It will be done in the following sections). As a continuation of the current section, we compared the CG-representation of protein with that in AA-representation.

Once established, a reliable 3D structure for MGD1 remains the task to evaluate the proper computational protocol to treat the protein at the CG level. Three used CG models show residue flexibility in good agreement with AA simulations (Fig. S3.10). Two approaches, namely ElNeDyn and Go, which impose additional restraints between pair of residues and aim at keeping the integrity of spatial protein structure, are widely used within the Martini force field. ElNeDyn incorporates the other harmonic bonds of a specific spring constant between pairs of residues to create unbreakable harmonic bonds which hinder the study of large-scale motions in the protein. In Go model, the harmonic bonds are replaced with Lennard–Jones interactions based on the contact map of the native protein structure, which makes the model more useful for conformational sampling in both equilibria and pulling simulations. The flexibility of MGD1, calculated in ElNeDyn and Go models is in close agreement with AA simulations. Expectedly, the ElNeDyn model provides slightly lower residue flexibility than Go and AA models. Nevertheless, both ElNeDyn and Go models failed to reproduce the flexibility of the large loop. The Martini applied to a protein without constraints may lead to non-physical protein dynamics. Nevertheless, the diagram, rmsd vs residue number for Martini protein is similar to that for ElNeDyn, Go, and AA simulations. The difference is that the absolute rmsf values are approximately twice as large as those for AA simulations. As in two other CG models, the flexibility of the loop in CG model is not as large as in AA model and is comparable with other flexible parts of the protein which could be a limitation of CG water representation. Martini water beads do not bear charges, which makes interactions of the LOOP with the protein more favourable than in solution. The only mismatching with ElNeDyn and Go models is the enhanced flexibility of aa 496-510, which are located on the second interdomain linker connecting C-domain and the C-terminal helix bound to N-domain (see [6]). The location of other residues possessed rmsf values higher than the mean values; as shown in Figure 3.10. Summing up, in accordance with a previous report [5], all three CG models are capable of recapturing the protein dynamics and can be used for further analysis of the dynamics of protein bound to the membrane.

The root mean square deviation (rmsd), measured for Cα-atoms, along the trajectory, showed that protein in stable within ElNeDyn and Go with corresponding averaged deviation of 0.17±0.01 and 0.27±0.2 nm. The rmsd of protein without any constraints drifts slightly during 10 μs, varying about 0.99±0.14 nm.

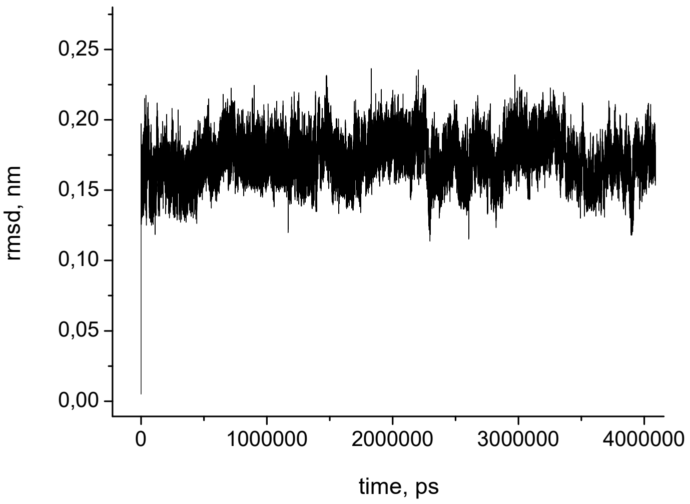

Figure S3.7. Rmsd values for backbone (BB) beads of MGD1 in water box calculated along CG-ElNeDyn MD trajectory.

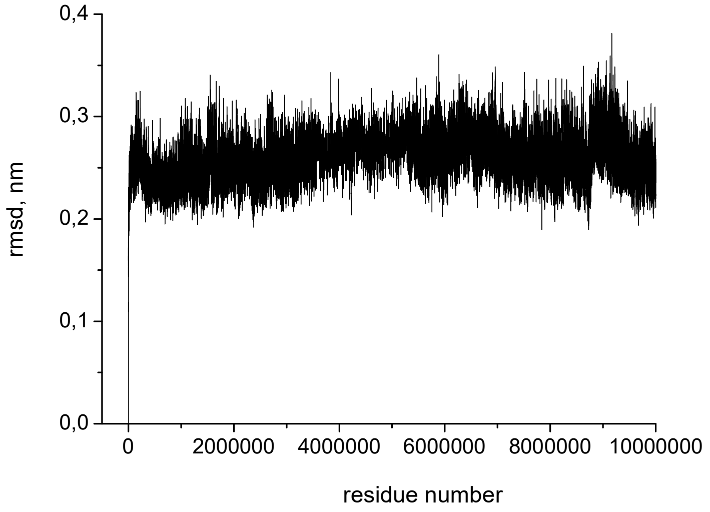

Figure S3.8. Rmsd values for backbone (BB) beads of MGD1 in water box calculated along CG-Go MD trajectory.

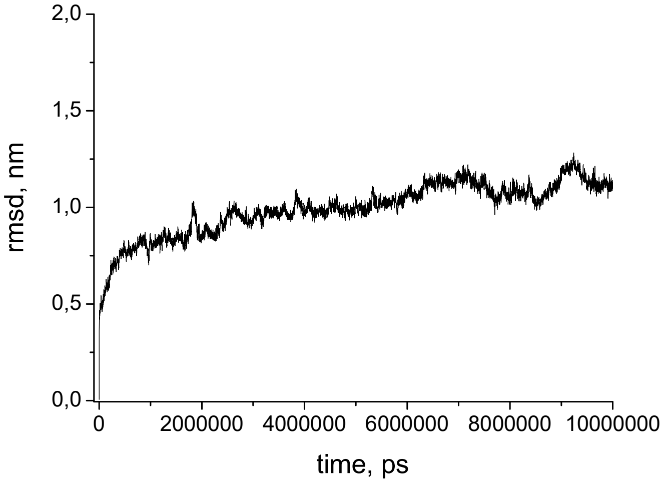

Figure S3.9. Rmsd values for backbone (BB) beads of MGD1 in water box calculated along CG MD trajectory.

RMSF

Rmsf values obtained from the Go model are in the closest agreement, among CG models, with those computed with the AA model. Nevertheless, the Go model fails to reproduce the flexibility of the LOOP. In the case of ElNeDyn constraint (where the large motions of the loop were not expected due to the incorporation of additional harmonic bonds of a certain spring constant between pairs of residues which create unbreakable harmonic bonds hinders the study of unfolding and folding processes), the values of rmsd are generally twice smaller than those calculated at AA and Go level of molecular representation. The rmsf values for the protein calculated with Martini without any restraints are approximately twice large than those for AA simulations. Interestingly, the flexibility of the loop in the CG model is not as large as in the AA model and is comparable with other flexible parts of the protein. Nevertheless, the contour of rmsd vs residue number is similar to that for AA simulations.

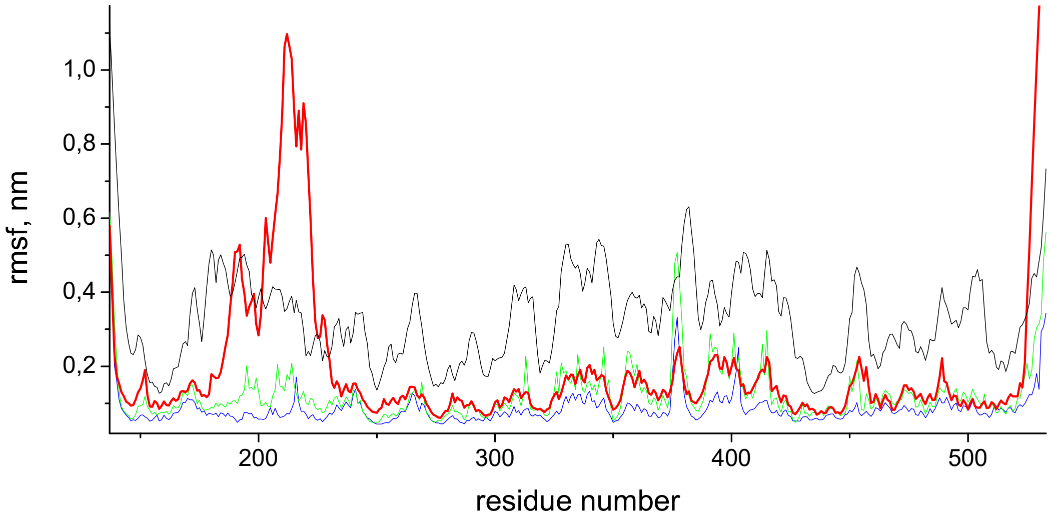

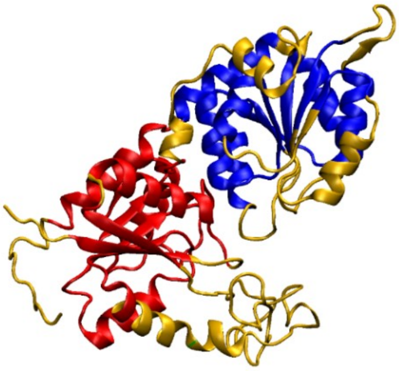

Figure S3.10. Rmsf values for Ca and backbone (BB) beads of MGD1 along AA (red) and CG MD trajectories, including ElNeDyn (blue), Go (green) and Martini without restraints (black) calculated for protein in a water box. The residues havingrmsf values higher than the mean value (calculated over all residues except the large loop region) are indicated with orange on the spatial structure of MGD1 (N- and C- domains are highlighted in red and blue, respectively).

S4. Orientation of the protein with respect to the membrane plane.

We used the PPM online service (https://opm.phar.umich.edu/ppm_server) to pre-orient the protein with respect to the membrane surface. The PPM server calculates rotational and translational positions of transmembrane and the peripheral protein in the membrane using their 3D structure (PDB coordinate file) as input. The protein is positioned in a lipid bilayer of adjustable thickness by minimizing its transfer energy from water to the membrane [7].

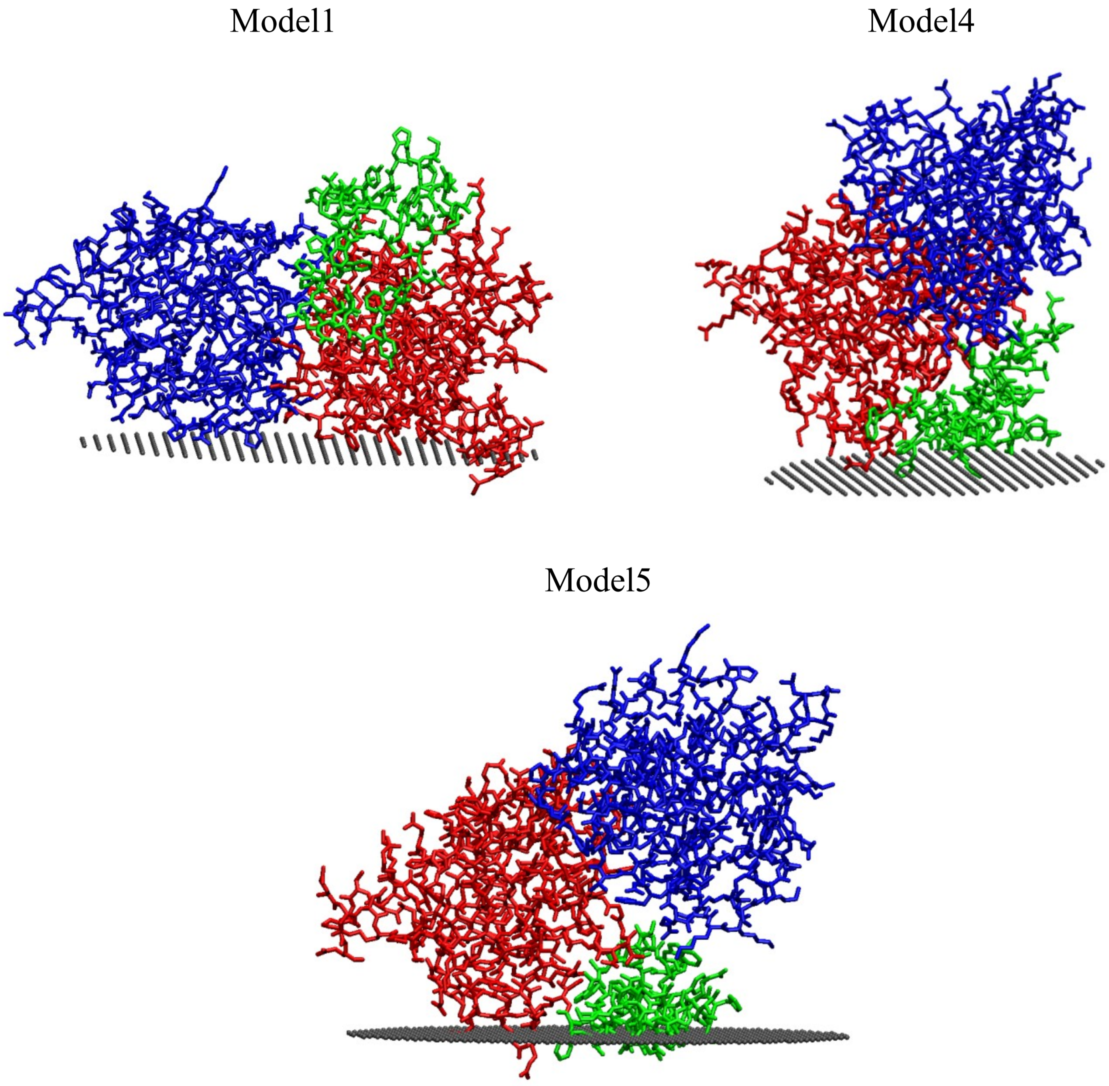

Figure S4.1. The orientation of three full sequence models of MGD1 molecule in respect to the membrane surface, indicated with grey dots. Color coding is following C-domain – blue, N-domain – red and the reconstructed loop within N-domain – green.

The output data from PPM are summarized in Table S4.1. In all the cases, the protein was predicted to be a peripheral one.

All the models can be partially embedded into the hydrophobic membrane with the energies of the transfer from water to the membrane varying from -3.2 to -5.6 kcal/mol. Model1 has the lowest energy of transfer; Model4 and Model5, have comparable energy values. In terms of interactions with the membrane, Model1 has favourable energy, the loop containing the residues essential for the catalysis is remote from the membrane. This orientation is not compatible with the catalysis to occur. Therefore, Model1 was excluded from consideration for the rest of the work.

In Models 4 and 5, the interactions with the membrane occur via the LOOP. The energy of transfer from water to the membrane is slightly higher (more favourable) for Model4 than that for Model5 despite the fact that the depth of embedding into the membrane is larger for Model5 than form Model4. Hence, both models were further studied in the complex with the explicit glycolipid bilayer.

Table S4.1. The orientation of Proteins Models in Membranes

	Depth/ Hydrophobic thickness, Å	ΔG transfer, kcal/mol	Tilt angle, °	Membrane embedded residues (in hydrocarbon core)
Model1	6.0 ± 1.7	-3.2	88	138,527,529,531-533
Model4	1.6 ± 2.1	-5.6	49	199,230
Model5	6.1 ± 1.2	-5.5	57	196-197,200,204-207,210,212,215-216,219,222-223, 225-226,230,233,240

S5. Protein – membrane interactions

Along the trajectory, the glycolipid membrane and the protein tend to form as many interactions as possible, which results in large distortion of the system on the large time-scale. This leads to two possible extreme cases. First, the membrane encapsulates the protein and finally forms an inverse phase. This is true for all CG approached used, namely standard Martini, ElNeDyn and Go. Second, the structure of the protein becomes destroyed and flattened, while the membrane keeps its planarity. The second possibility was observed only when the protein structure had no restraints. Note, that this behaviour was observed only when the glycolipids were added to the system. But this is not the case when only PG and DAG form the membrane. Such a behavior was never reported for some other monotopic proteins when the membrane consisted of dipalmitoylphosphatidylcholine (DPPC) lipids [8] or PG/DAG only membranes [9]. Thus, we observed that while gaining more flexibility due to the dynamics of unsaturated galactolipid tails, the system tends to increase the interaction energy between protein and membrane. We face a limitation of standard Martini to describe the interactions in our particular or similar system, e.g. when the monotopic proteins and the membranes contain a large number of lipids with unsaturated tails and hydrophilic bulk head groups, at large time-scales (more than 10 μs). On the other hand, the pure membrane and the M4-like membrane with transmembrane proteins inserted [10] remained planar *in silico*. One may assume that *in vivo*, the galactolipid bilayer has additional stabilization from transmembrane proteins. The variations in local lipid concentration may induce a preference for lateral or hexagonal phase and thus regulate the membrane flexibility. However, the lipid clustering is fast and rapidly reach the equilibration. In CG simulations, starting from a random lipid distribution, the spontaneous cluster formation occurred repetitively within dozens of nanoseconds. The protein-induced lipid re-organization takes hundreds of nanoseconds [9]. Thus, the described effects of lipid assembly took place since the beginning of trajectories. Upon interactions with MDG1 the membrane analyzed the curvature was comparable with that without protein.

Table S5.1. Number of contacts between protein and lipid species on the distance within 0.6 nm along the trajectory.

	POPG	PODG	MGDG	DGDG
M1	12 ± 4	31± 8		
M4	12 ± 13	22 ± 4	310 ± 72	175± 15

Table S5.2. The list of the shortest distances DAG with MGD1 protein averaged over the trajectory (nm) and the minimal values (nm) reached during the trajectory.

	Residue	Average	Dev	Min
1.	H185	0.48	0.018	0.42
2.	P187	0.48	0.019	0.42
3.	W188	0.48	0.024	0.42
4.	D184	0.49	0.028	0.42
5.	F199	0.50	0.053	0.42
6.	F190	0.51	0.060	0.43
7.	K202	0.51	0.065	0.42
8.	T186	0.51	0.044	0.44
9.	P189	0.51	0.084	0.42
10.	T151	0.53	0.066	0.43
11.	T229	0.57	0.120	0.44
12.	R195	0.60	0.159	0.43
13.	F230	0.62	0.165	0.43
14.	S196	0.64	0.176	0.43
15.	N223	0.66	0.190	0.43
16.	S222	0.66	0.179	0.44
17.	W182	0.68	0.175	0.42
18.	G152	0.73	0.076	0.48
	H155	1.61	0.09	1.01
	R156	1.48	0.10	0.95

Table S5.3. The list of the shortest distances POPG with MGD1 protein averaged over the trajectory (nm) and the minimal values (nm) reached during the trajectory.

	Num	Average	Dev	Min
1.	R195	0.50	0.037	0.43
2.	R263	0.54	0.119	0.43
3.	K269	0.55	0.122	0.42
4.	F190	0.56	0.136	0.42
5.	S222	0.57	0.156	0.43
6.	T151	0.62	0.194	0.44
7.	V219	0.63	0.198	0.42
8.	R260	0.65	0.212	0.43
9.	L268	0.65	0.208	0.40
10.	Q192	0.65	0.173	0.43
11.	D184	0.66	0.167	0.44
12.	R233	0.66	0.285	0.43
13.	W188	0.70	0.237	0.43
14.	S264	0.72	0.214	0.44
15.	W182	0.74	0.253	0.43
16.	G266	0.76	0.226	0.44
17.	F230	0.78	0.275	0.42
18.	H185	0.80	0.202	0.44
19.	P189	0.80	0.210	0.43
	H155	1.23	0.352	0.45
	H156	0.98	0.278	0.46

REFERENCES

[1] Eyal, E., Lum, G. & Bahar, I. The anisotropic Network Model web server at 2015 (ANM 2.0). *Bioinformatics* **31,** 1487–1489 (2015).

[2] Marrink, S. J., Risselada, H. J., Yefimov, S., Tieleman, D. P. & de Vries A. H., The MARTINI force field: coarse grained model for biomolecular simulations. *J. Phys. Chem. B*, **111,** 7812–7824 (2007).

[3] Periole, X., Cavalli, M., Marrink, S. J. & Ceruso, M. A. Combining an elastic network with a coarse-grained molecular force field: structure, dynamics, and intermolecular recognition. *J.*

Chem. Theory Comput. **5,** 2531–2543 (2009).

[4] Poma, A.B., Cieplak, M. & Theodorakis, P. E. Combining the MARTINI and structure-based coarse-grained approaches for the molecular dynamics studies of conformational transitions in proteins. *J. Chem. Theory Comput.* **13,** 1366–1374 (2017).

[5] Lu, Y. & Salsbury Jr., F. R. Recapturing the correlated motions of protein using coarse-grained models. *Protein Pept. Lett.* **22,** 654–659 (2015).

[6] Rocha, J. *et al.* Structural insights and membrane binding properties of MGD1, the major galactolipid synthase in plants, *Plant J.*, **85,** 622–633 (2016).

[7] Lomitze, M. A., Pogozheva I. D., Joo, H., Mosberg, H. I. & Lomize, A. L. OPM database and PPM web server: resources for positioning of proteins in membranes. *Nucleic. Acids Res.*, **40** D370–6 (2012).

[8] Balali-Mood, K., Bond P. J. & Sansom, M. S. P. Interaction of monotopic membrane enzymes with a lipid bilayer: A coarse-grained MD simulation study, *Biochemistry*, **48,** 2135–2145 (2009).

[9] Nitenberg, M. *et al.* Mechanism of activation of plant monogalactosyldiacylglycerol synthase 1 (MGD1) by phosphatidylglycerol. *Glycobiology*, **30**, 396-406, (2020).

[10] F.J. van Eerden, T. van den Berg, P.W.J.M. Frederix, D.H. de Jong, X. Periole, S.J. Marrink. Molecular dynamics of photosystem II embedded in the thylakoid membrane. *JPCB*, **121:**3237–3249 (2017).
